# Supplementary material for: Validation of human telomere length multi-ancestry meta-analysis association signals identifies POP5 and KBTBD6 as human telomere length regulation genes
Source: Nat Commun. 2024 May 24;15:4417. doi: 10.1038/s41467-024-48394-y (PMC11126610; doi:10.1038/s41467-024-48394-y)
Supplement: Supplementary file 1 — Supplementary Information [file 41467_2024_48394_MOESM1_ESM.pdf]

Validation of human telomere length multi-ancestry meta-analysis association signals identifies *POP5* and *KBTBD6* as novel human telomere length regulation genes

Keener et al.

Supplementary Information

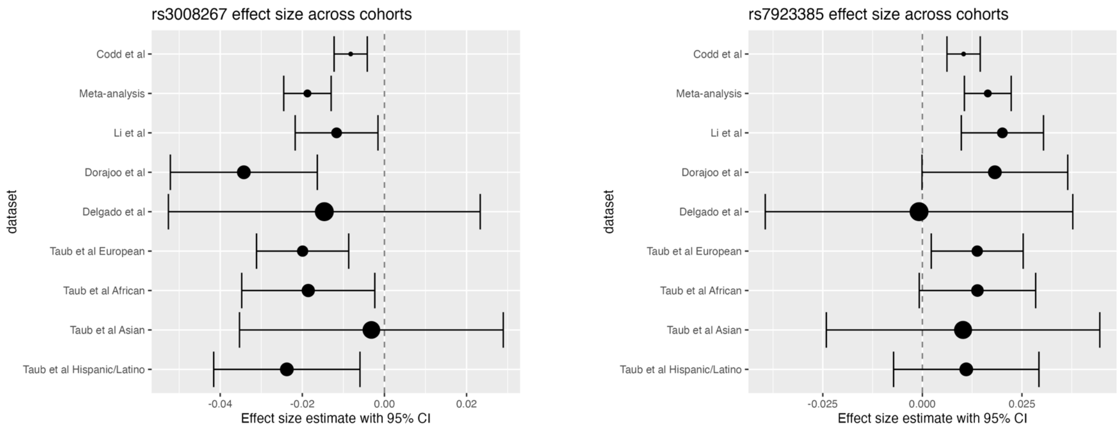

Supplementary Figure 1: Replication of novel signals in an independent telomere length GWAS.
Comparison of lead SNP effect size estimates at two novel signals in our analysis. The error bars represent the 95% confidence interval for the effect size estimate and the size of the dot represents the standard error of the effect estimate. We compared the seven meta-analysis input datasets to an independent telomere length GWAS (Codd et al. 2021). Codd et al. N=472,174; Meta-analysis N=211,369; Li et al. N=78,592; Dorajoo et al. N=23,096; Delgado et al. N=5,075; Taub et al. European N=51,654; Taub et al. African N=29,260; Taub et al. Asian N= 5,683; Taub et al. Hispanic/Latino N=18,019. Source data are provided as a Source Data file.

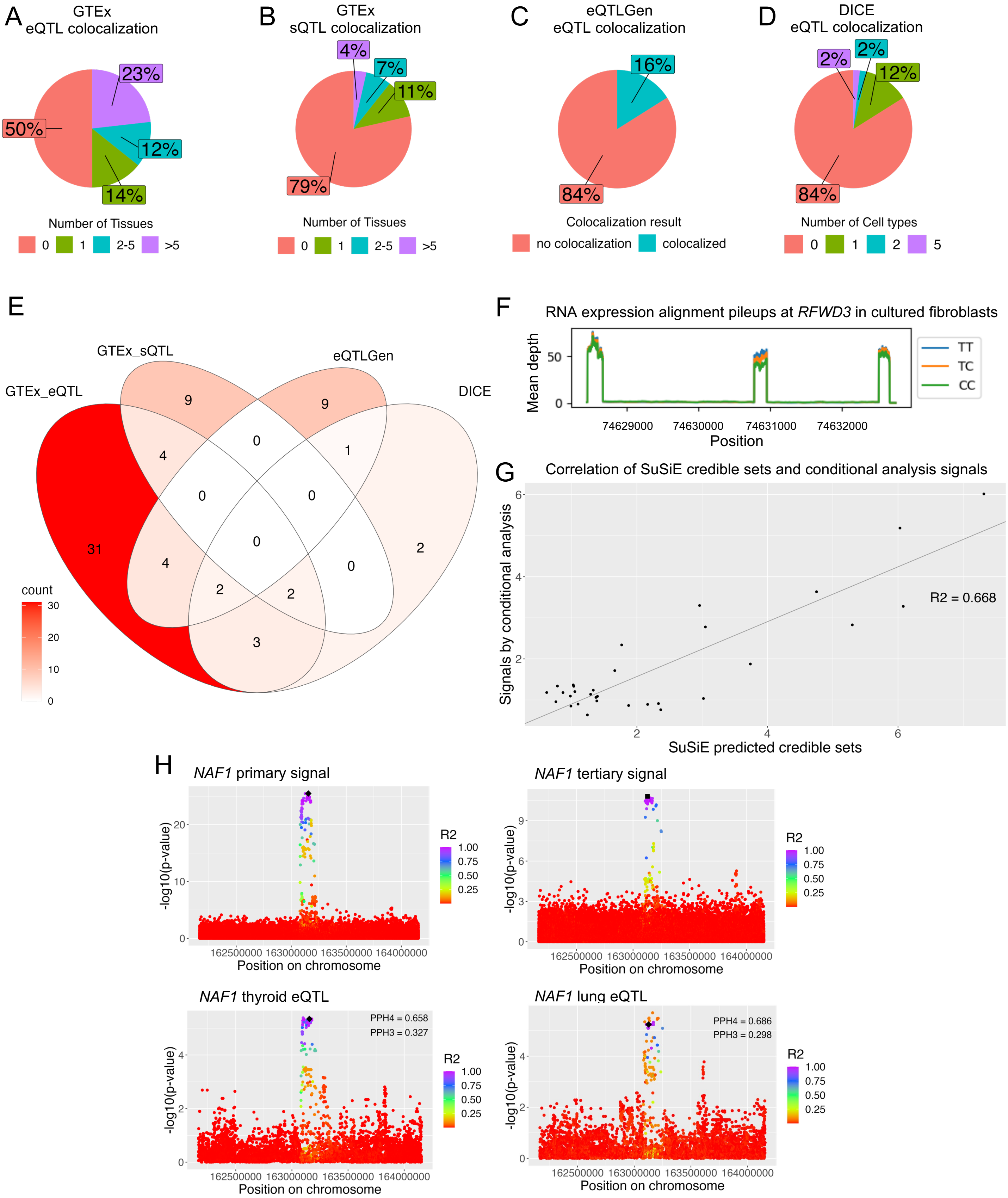

Supplementary Figure 2: Fine-mapping analyses nominate putative causal variants and genes affecting telomere length. A-B. Percent of meta-analysis signals that colocalize (PPH4 > 0.7) with a GTEx cis-eQTL or cis-sQTL for any gene across differing numbers of tissues. C. Percent of meta-analysis signals that colocalize (PPH4 > 0.5) with a DICE eQTL for any gene in any cell type. The threshold for PPH4 was reduced because the DICE dataset has lower power to detect eQTLs since the dataset is derived from 91 individuals. A-C. In some instances one signal may colocalize with one gene in tissue/cell type X while colocalizing with a second gene in tissue/cell type Y; this case would be reported as number of tissue/cell type = 2. D. Percent of meta-analysis signals that colocalize (PPH4 > 0.7) with an eQTLGen cis-eQTL. eQTLGen cis-eQTLs are derived from whole blood only. E. Venn diagram showing in which datasets meta-analysis signals colocalized with the same gene quantitative trait locus (QTL) in any cell type across datasets. F. RNA expression pileup plots from GTEx v8 for *RFWD3* in cultured fibroblasts. The plot is stratified by genotype for the sentinel SNP at the meta-analysis locus. G. Correlation of the number of SuSiE predicted credible sets and the number of signals by conditional analysis (Taub et al. 2022). Source data are provided as a Source Data file. H. Manhattan plot for the *NAF1* primary signal from the TOPMed pooled GWAS analysis colored by r^2 with the lead SNP (black diamond). The best colocalization result for this signal was the *NAF1* eQTL in thyroid (Manhattan plot shown). After two rounds of conditional analysis on the lead SNP and secondary signal lead SNP at the *NAF1* locus, a tertiary signal remained significant (Taub et al. 2022). The Manhattan plots for the tertiary signal and *NAF1* lung eQTL are colored by r^2 with the lead SNP at the tertiary signal (black square). The best colocalization result for the tertiary GWAS signal was with the *NAF1* eQTL in lung. The *NAF1* eQTL in thyroid did not colocalize with the *NAF1* eQTL in lung (PPH3 = 0.721, PPH4 = 0.217). Source data are provided as a Source Data file.

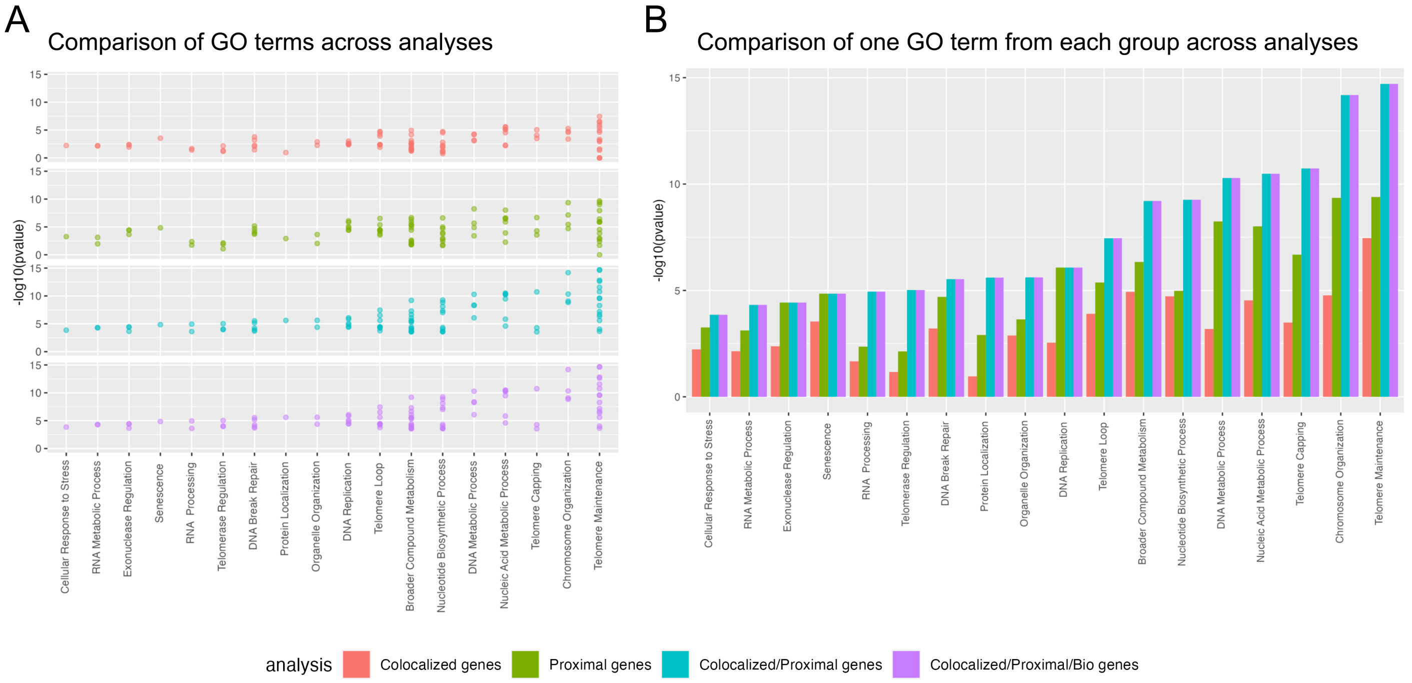

Supplementary Figure 3: Comparison of GO enrichment analysis results with different gene input datasets. A-B. Each meta-analysis locus was assigned a gene based on genes indicated by colocalization analysis (red), the proximal gene (green), genes indicated by colocalization analysis where possible and proximal genes where not possible (blue), or genes indicated by proximity-plus-knowledge, colocalization analysis, or proximal genes where no other information was available (purple). In the fourth case (purple) there were five loci where a nearby gene has known roles in telomere length regulation but was neither the proximal gene nor the gene indicated by colocalization analysis (further explored in the Supplemental Note). Note that there are more genes included in the proximal gene list than the colocalized gene only list as every meta-analysis signal has a proximal gene but not all have colocalization results. Enrichment was calculated using PANTHER and a Fisher's exact test with false discovery rate correction. A. GO terms were manually grouped based on related biology. All GO terms that had FDR < 0.05 in at least one analysis are shown. Group assignments and comparison of enrichment for all GO terms with FDR < 0.05 are reported in Supplementary Table 8. B. The GO term with the smallest pvalue in the Colocalized+Proximal+Bio analysis was chosen for each group and the comparison of pvalues across analyses are shown.

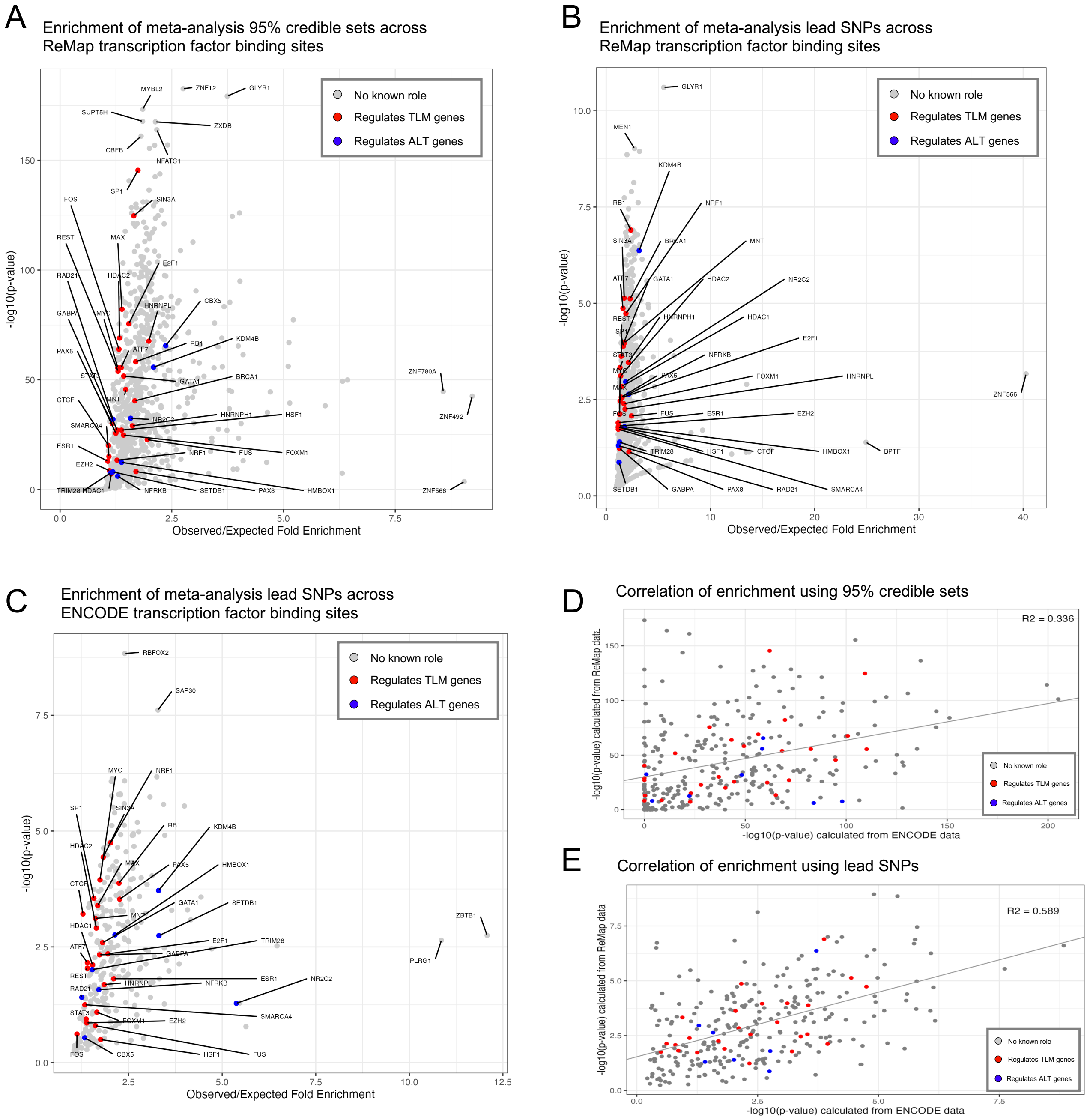

Supplementary Figure 4: Meta-analysis signals are enriched for transcription factor binding sites of transcription factors with roles in telomere length regulation.

The red points represent transcription factors with known roles in regulating telomere length maintainence (TLM) genes and the blue points represent transcription factors with known roles in the alternative telomere lengthening (ALT) pathway. In all cases enrichment was calculated using a one-sided binomial test (Methods). A. The enrichment of 95% credible set SNPs across all transcription factors with data available from ReMap data (Methods). There were 1,034 transcription factors plotted (28 red, 8 blue, 998 grey). There were 176 transcription factors that fell at the (0,0) coordinate and are not shown for clarity; one (XRCC3) had known roles in ALT. B. The enrichment of only the lead SNP at each meta-analysis signal across all transcription factors with data available from ReMap data (Methods). There were 950 transcription factors plotted (28 red, 7 blue, 915 grey). There were 196 transcription factors that fell at the (0,0) coordinate and are not shown for clarity; one (XRCC3) had known roles in ALT. C. The enrichment of only the lead SNP at each meta-analysis signal across all transcription factors with data available from ENCODE data (Methods). There were 308 transcription factors (25 red, 8 blue, 275 grey). There were 22 transcription factors that fell at the (0,0) coordinate and are not shown for clarity; one (XRCC3) had known roles in ALT. D-E. The enrichment of transcription factors included in both the ReMap and ENCODE datasets are shown. The grey line represents the regression between these two variables and the R2 is shown in the top right corner. Source data for Supplementary Figure 4 are provided as a Source Data file.

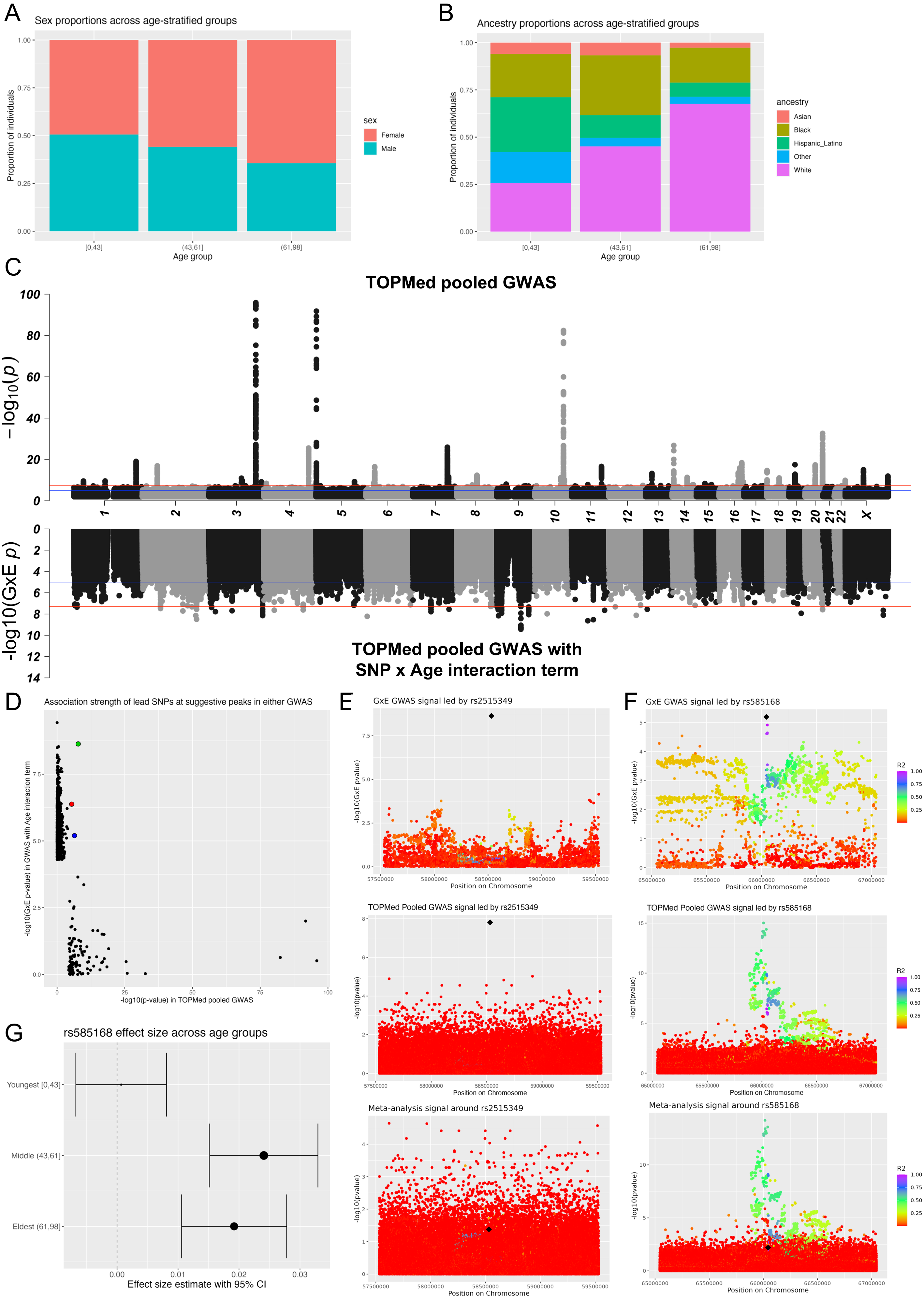

Supplementary Figure 5: Demographics for age-stratified telomere length GWAS.

A-B. The 109,122 TOPMed individuals with telomere length estimates (Taub et al. 2022) were divided into three age groups such that there was a similar number of individuals per group. There were 36,980 individuals in the [0,43] group, 37,470 individuals in the (43,61] group, and 34,671 individuals in the (61,98] group. A. The proportion of individuals of each biological sex in each age group. B. The proportion of individuals of different ancestries in each age group. Ancestry was previously determined computationally (Taub et al. 2022). C. Miami plot displaying the summary statistics of the TOPMed pooled GWAS (Taub et al. 2022) (top, SNPs with p-values < 0.01) and the same GWAS but with a SNP x age interaction term added (bottom). The blue horizontal line is a suggestive threshold ($p<5x10^{-5}$) and the red line is a genome-wide significant threshold ($p<5x10^{-8}$). D. Each point is the lead SNP at a signal was suggestive ($5x10^{-5}$) in either the TOPMed pooled GWAS or the TOPMed pooled GWAS with an interaction term between genotype and age. The red point is rs8012195, the lead SNP at the signal near *TCL1A, t*he green point is rs2515349, and the blue point is rs585168. Source data are provided as a Source Data file. E. Manhattan plots showing the region around rs2515349 in the pooled TOPMed GWAS with an age x genotype effect, in the pooled TOPmed GWAS, and in the meta-analysis. F. Manhattan plots showing the region around rs585168 in the pooled TOPMed GWAS with an age x genotype effect, in the pooled TOPmed GWAS, and in the meta-analysis. G. Forest plot showing the effect size estimates for rs585168 in the age-stratified GWAS. Source data are provided as a Source Data file.

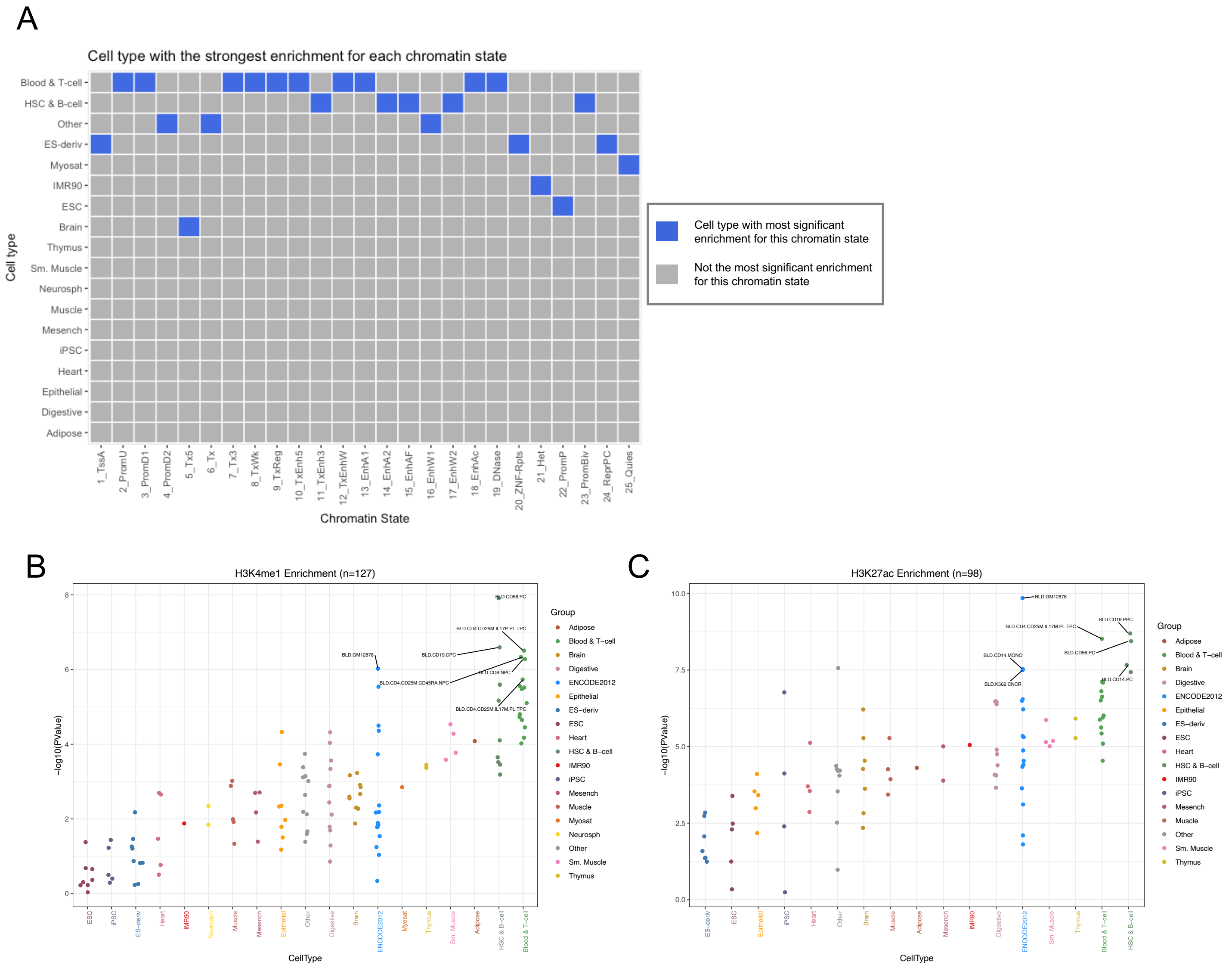

Supplementary Figure 6: Enrichment of chromatin marks or states from Roadmap Epigenomics across cell types.
A. Meta-analysis of cell type samples enrichment for each chromatin state (Figure 5A). Enrichment was calculated using a Fisher's method to calculate a combined chi-squared statistics for the samples in each cell type. The cell type with the most significant enrichment is indicated in blue. For more information on the interpretation of the 25 state chromatin model, see the Roadmap Epigenomics publication or link in Methods. B-C. Enrichment of Roadmap cell types for sentinel SNPs in H3K4me1 (A) or H3K27ac (B) peaks across 127 and 98 cell types, respectively. Included samples are listed in Supplementary Table 13.

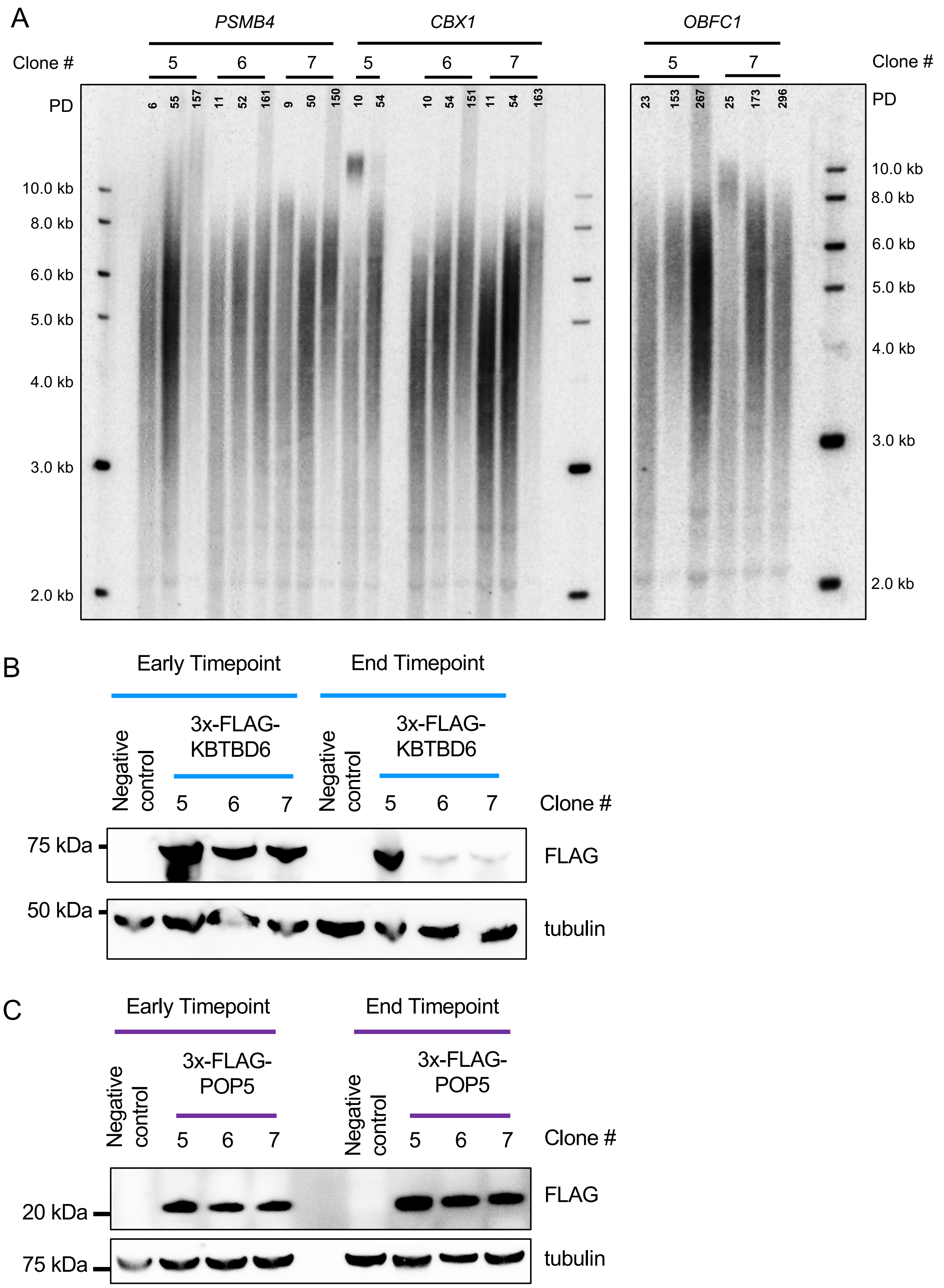

Supplementary Figure 7: Control data for overexpression of *KBTBD6* and *POP5*.
A. *PSMB4*, *CBX1*, or *OBFC1* was constitutively overexpressed from the CMV promoter in HeLa-FRT cells using the FLP-in system. Telomere Southern blots showing the bulk telomere length from a population of cells following an approximate normal distribution. Molecular weight standards were run alongside the samples and their size is indicated in kilobases (kb). Three time points are shown for each clone and the estimated number of population doublings (PD) for each timepoint are indicated. Three clones were observed for each gene with trends similar to those shown here. All transfection experiments began from the same population of HeLa-FRT cells. B. KBTBD6 overexpression was maintained in clone 5 over time but was lost in clones 6 and 7 as demonstrated by the end timepoint. The early timepoint was passage 8 of the experiment, approximate population doublings were: clone 5 = 51, clone 6 = 33, clone 7 = 45. The end timepoint was passage 31, approximate population doublings were: clone 5 = 273, clone 6 = 257, clone 7 = 274. C. POP5 overexpression was maintained across all three clones. The early timepoint was passage 8 of the experiment, approximate population doublings were: clone 5 = 65, clone 6 = 59, clone 7 = 67. The end timepoint was passage 31 of the experiment, approximate population doublings were: clone 5 = 296, clone 6 = 297, clone 7 = 318. Source data for Supplementary Figure 7 are provided as a Source Data file.

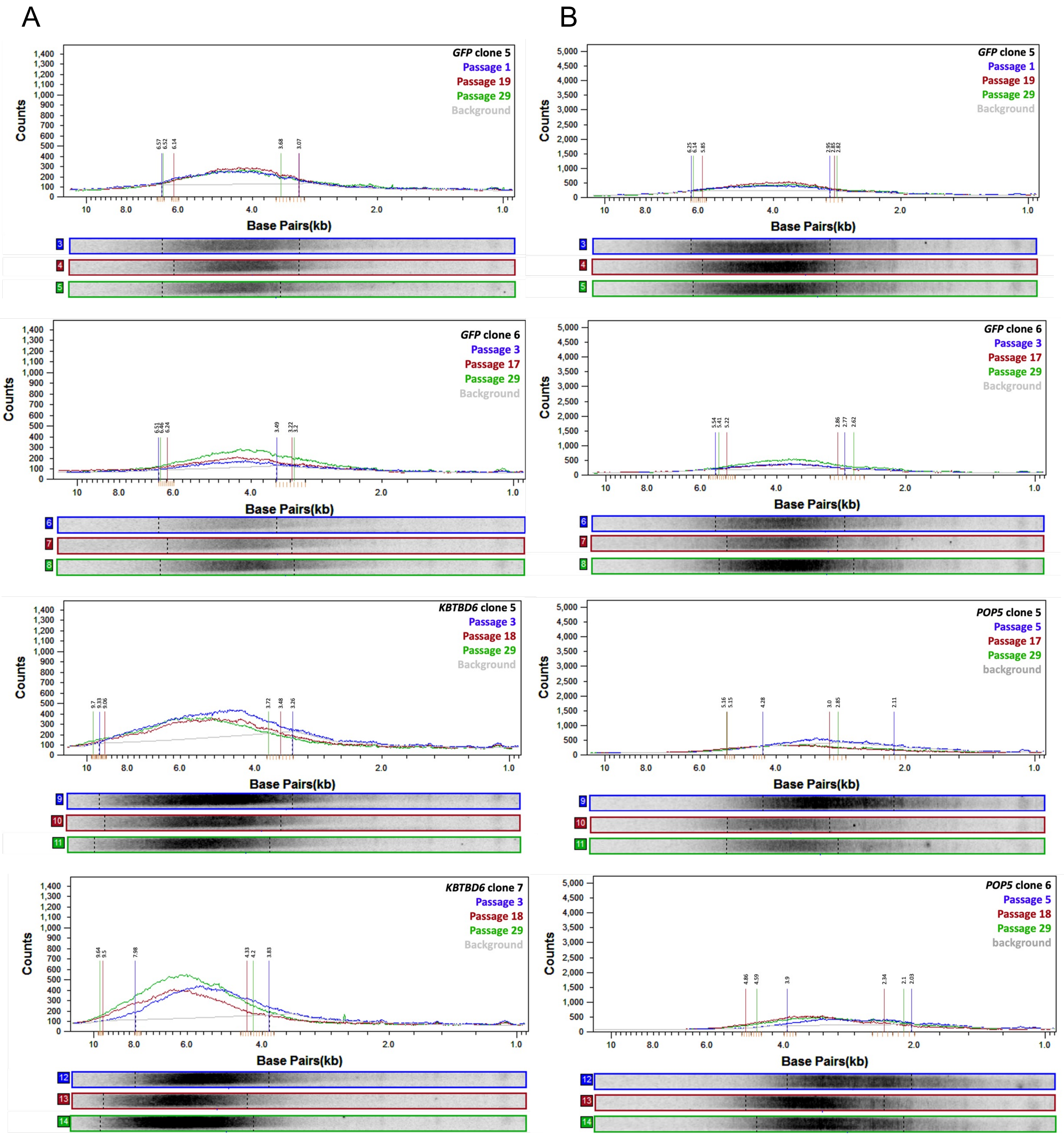

Supplementary Figure 8: ImageQuant TL estimation of minimum, median, and maximum telomere length. Unprocessed scans of the telomere Southern blots were imported into ImageQuant TL and median telomere length was calculated taking molecular weight markers on either side of the Southern into account. The median telomere length was automatically estimated as the maximum value in these line plots for each line. Line plots were generated for the three time points (timepoints indicated by line color) for each clone. The grey lines indicate the background signal estimated by ImageQuant TL. The Southern blot lanes analyzed in each plot are shown below their respective line plots. The software indicates the range of the signal that it takes into account when estimating the median and these boundaries (dotted lines on the lanes) were used to represent the minimum and maximum telomere lengths. The vertical lines on the line plot were added manually and colored to match the sample they estimate, the values above them represent the estimated minimum or maximum. The software does not provide a quantitative estimate of these boundaries and so we inferred them from the units on the x-axis. Where the minimum or maximum did not fall close to an automated tick mark, we imputed additional tick marks (orange) by anchoring two lines on the available tick marks and adding another three lines in between, then distrubted evenly horizontally using Microsoft PowerPoint. A. Line plots from Figure 6A. B. Line plots from Figure 6B.

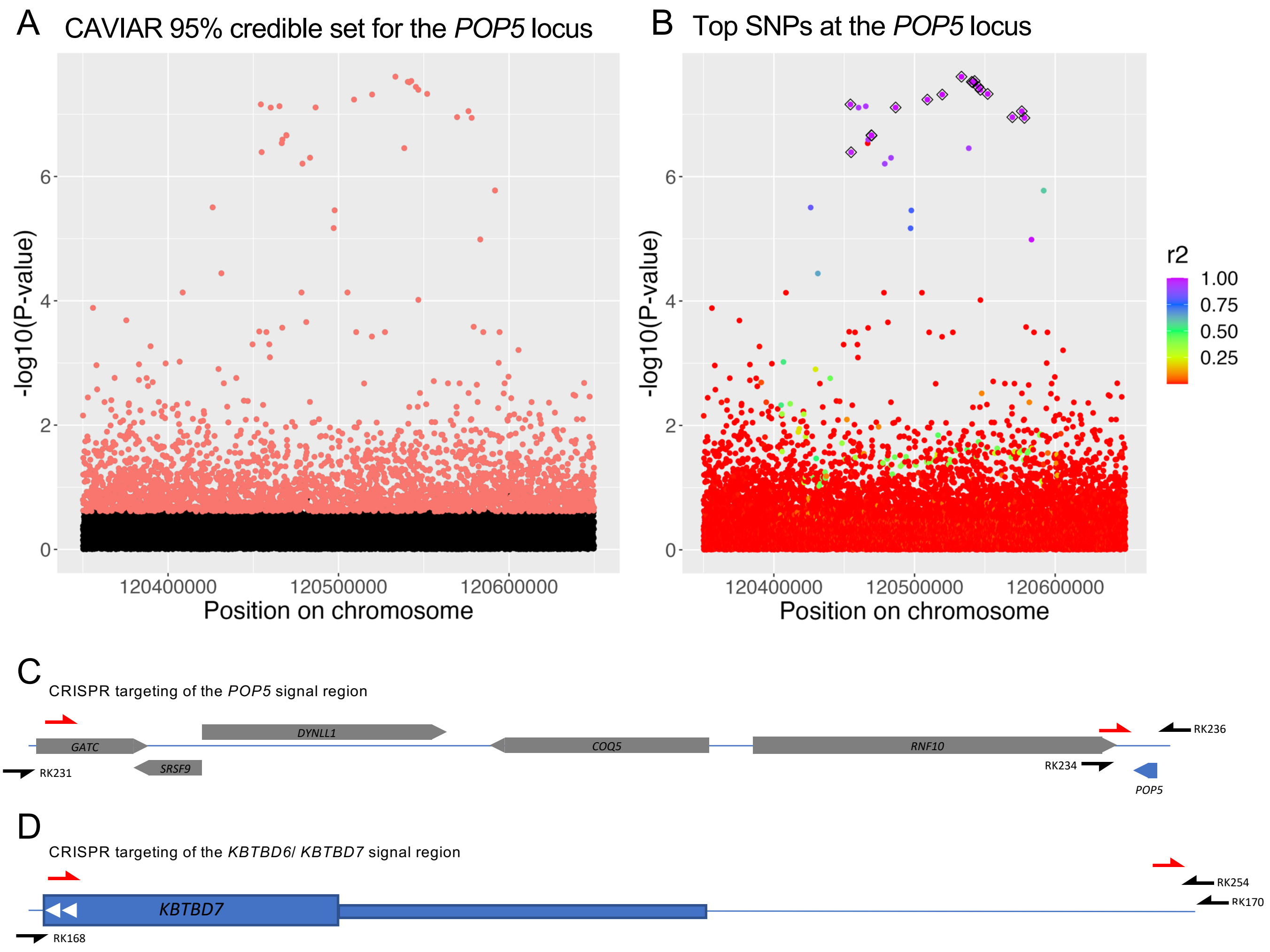

Supplementary Figure 9: CRISPR/Cas9 targeted regions.
A. Manhattan plot showing the association signal near *POP5*. Red SNPs were in the CAVIAR 95% credible set. CAVIAR was run assuming there was one causal SNP in the signal (c=1). Source data are provided as a Source Data file. B. Manhattan plot showing the association signal near *POP5*. Color indicates linkage disequilibrium (r^2) calculated with respect to the lead SNP. Source data are provided as a Source Data file. C. 124 kb region targeted for CRISPR/Cas9 editing within the *POP5* association signal region. The red half arrows indicate the position of CRISPR/Cas9 gRNA sequences. The black half arrows indicate the position of primers used to genotype CRISPR/Cas9-edited cells (Methods). Primer and guide sequences are reported in Supplementary Table 14. The position and size of the indicated coding sequences were taken from the UCSC genome browser and are to scale. *POP5* is indicated in blue. D. 938 bp ATAC-seq peak region targeted for CRISPR/Cas9 editing within the *KBTBD6*/ *KBTBD7* association signal region. The red half arrows indicate the position of CRISPR/Cas9 gRNA sequences. Primer and guide sequences are reported in Supplementary Table 14. The position and structure of the *KBTBD7* coding sequence was taken from the UCSC genome browser and is to scale.

Supplementary Note 1

Supplementary Note 1 conveys the rationale for the assigned putative causal gene for each signal. For 15 signals no colocalization results were available and there were no known genes involved in telomere length in the region (Supplementary Note Table 1, see rows with “Proximal gene (no other supporting information)”). In these cases, the proximal gene was assigned. For 33 signals there was colocalization data in at least one QTL dataset (Figure 2A). However, colocalization within and between datasets supported different genes for 12 meta-analysis signals (Discussed below). Furthermore, TWAS analysis identified had significant results for 19 meta-analysis signals, nine of which had some evidence of colocalization and seven of which agreed with the putative causal gene nominated by colocalization analysis. In addition, TWAS identified nine genes as associated with telomere length (*BECN1*, *CMYA5*, *MAN1B1*, *LIME1*, *MPHOSPH9*, *SNTB2*, *SNX17*, *SPI1*, *TRAP1*), but these genes are not in within 100 megabases of any genome-wide significant loci. For each signal we show the Manhattan plot for the meta-analysis signal and the best colocalization result for each gene in the dataset where colocalization was strongest (highest PPH4). We show all instances where PPH4 > 0.5 but we consider this threshold to be moderate, a higher threshold of PPH4 > 0.7 should be considered as strongly colocalized. For instances where there was strong colocalization (PPH4 > 0.7) with a GTEx sQTL we show the RNA pileup plot. All colocalization results are reported in Supplementary Data 4-7. The meta-analysis Manhattan plots were centered on the lead SNP and include the region ± 1 megabase the lead SNP (hg38) and the x-axis is matched for each plot. In all plots the meta-analysis lead SNP was shown as a black diamond and r^2 was calculated with respect to the lead SNP using all TOPMed individuals included in the meta-analysis.

Supplementary Note Table 1:

Lead SNP	Novel Signal	Attributed gene	Supporting evidence for attributed gene
rs542948485		DCLRE1B	Known biology, colocalization
rs12044242		PSMB4	Colocalization, proximal gene
rs146042055		PARP1	Known biology
rs62139251		TSPYL6	Colocalization
rs11894326		CPS1	Colocalization
rs35510081		TERC	Known biology
rs3775946		SLC2A9	Colocalization, proximal gene
rs4691895		NAF1	Known biology, colocalization
rs33961405		TERT	Known biology
rs56099285		UBE2D2	Proximal gene (no other supporting information)
rs3131064		POU5F1	Colocalization
rs1150748		BAG6	Colocalization

rs6968500		POT1	Known biology
rs10954213		IRF5	Colocalization, TWAS, proximal gene
rs3008267	*	ZNF596	Proximal gene (no other supporting information)
rs10958468		TMEM68	Proximal gene (no other supporting information)
rs73687065		TERF1	Known biology, proximal gene
rs10111287		VIRMA	Colocalization, proximal gene
rs62560860		IRL11A	Colocalization
rs958919990	*	GRHPR	Proximal gene (no other supporting information)
rs3736462		TASOR2	Proximal gene (no other supporting information)
rs3758526		NOC3L	Proximal gene (no other supporting information)
rs7923385	*	RRP12	TWAS, Proximal gene
rs11190126		NKX2-3	Proxmal gene (no other supporting information)
rs112519582	*	BTRC	Proximal gene (no other supporting information)
rs2475215		OBFC1	Known biology
rs12241155		SORCS1	Proximal gene (no other supporting information)
rs582297		ATM	Known biology, Colocalization, Proximal gene
rs74892322		POP5	Known biology
rs28755851		ZCCHC8	Known biology
rs1411041		KBTBD6/KBTBD7	Colocalization, TWAS, Proximal gene
rs532687339		TINF2	Known biology
rs4902358		MAX	Colocalization, proximal
rs2572		DCAF4	Proximal gene (no other supporting information)
rs11623533		PPP4R3A	Proximal gene (no other supporting information)
rs2887399		TCL1A	Colocalization, TWAS, proximal gene
rs113119217		ATP8B4	Colocalization, TWAS, proximal gene
rs12934863		DUS2	Colocalization, proximal gene
rs9939870		TERF2	Known biology, colocalization, TWAS, proximal gene
rs12149396		CLEC18C	Colocalization
rs7193541		RFWD3	Colocalization, proximal gene
rs6564996		MPHOSPH6	Colocalization, proximal gene
rs11117354		BANP	Proximal gene (no other supporting information)
rs59922886		CTC1	Known biology, colocalization, proximal gene
rs208011	*	CBX1	Known biology

rs144204502		TK1	Colocalization, proximal gene
rs2124616		TYMSOS	Colocalization
rs28782011		SETBP1	Proximal gene (no other supporting information)
rs139955496		POLI	Proximal gene (no other supporting information)
rs8105767		ZNF257	Colocalization, proximal gene
rs79476302		SAMHD1	Proximal gene (no other supporting information)
rs114703330		RTEL1	Known biology, colocalization, proximal gene
rs28663120		GAB4	Colocalization, proximal gene
rs131784		TYMP	Colocalization, TWAS
rs12394264		MIR223HG	Colocalization, proximal gene
rs5945232		DKC1	Known biology

rs542948485 (chr1:113917053:G:T)

	rsid	hgnc_symbol	dataset	tissue	PPH3	PPH4
1	rs542948485	DCLRE1B	eQTLGen	Whole_Blood	0.2104468	0.7707323

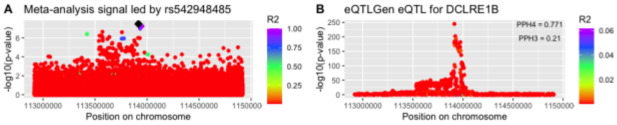

This meta-analysis signal colocalized with a *DCLRE1B* eQTL in eQTLGen. *DCLRE1B* is also the proximal gene. In addition, *DCLRE1B* is known to contribute to telomere length regulation. Therefore, we concluded that *DCLRE1B* was the best supported putative casual gene.

rs12044242 (chr1:151398465:C:T)

	rsid	hgnc_symbol	dataset	tissue	PPH3	PPH4
1	rs12044242	PRUNE1	GTEx_eQTL	Lung	0.43129704	0.5425956
2	rs12044242	SELENBP1	GTEx_eQTL	Thyroid	0.09401444	0.9058028
3	rs12044242	POGZ	GTEx_eQTL	Cells_Cultured_fibroblasts	0.17356127	0.8261163
4	rs12044242	PSMB4	GTEx_eQTL	Nerve_Tibial	0.24215216	0.7236395
5	rs12044242	PSMB4	eQTLGen	Whole_Blood	0.22084058	0.7786861

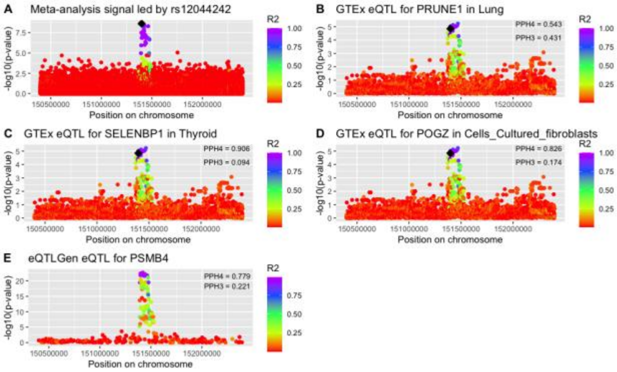

This meta-analysis signal colocalized with QTLs for *PRUNE1*, *SELENBP1*, *POGZ*, and *PSMB4*. TWAS nominated a fifth gene, *SLC39A1*. We observed that the *PSMB4* eQTL colocalization was replicated in eQTLGen (table). *PSMB4* is also the proximal gene for this signal, therefore we concluded that *PSMB4* was the best supported putative causal gene.

rs62139251 (chr2:54251468:G:T)

	rsid	hgnc_symbol	dataset	tissue	PPH3	PPH4
1	rs62139251	TSPYL6	GTEx_eQTL	Testis	0.02586353	0.9741365

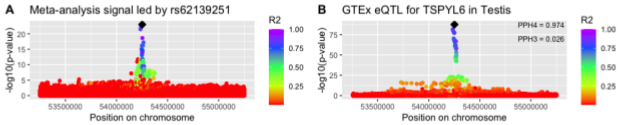

This meta-analysis signal only colocalized with a *TSPYL6* eQTL. The proximal gene was *ACYP2.* We concluded that *TSPYL6* was the best supported putative causal gene.

rs11894326 (chr2:209808365:C:T)

	rsid	hgnc_symbol	dataset	tissue	PPH3	PPH4
1	rs11894326	CPS1	GTEx_eQTL	Cells_EBV-transformed_lymphocytes	0.09700785	0.8533625

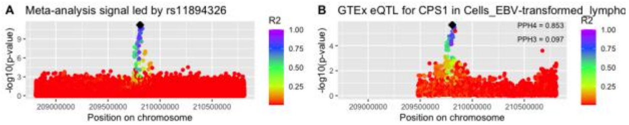

This meta-analysis signal only colocalized with a *CPS1* QTL. The proximal gene was *UNC80.* We concluded that *CPS1* was the best supported putative causal gene.

rs3775946 (chr4:9993632:A:G)

	rsid	hgnc_symbol	dataset	tissue	PPH3	PPH4
1	rs3775946	SLC2A9	GTEx_sQTL	Lung	0.1054985	0.8939474

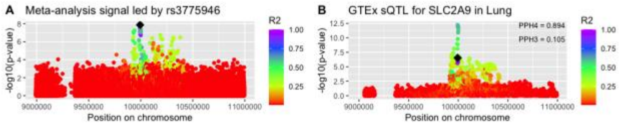

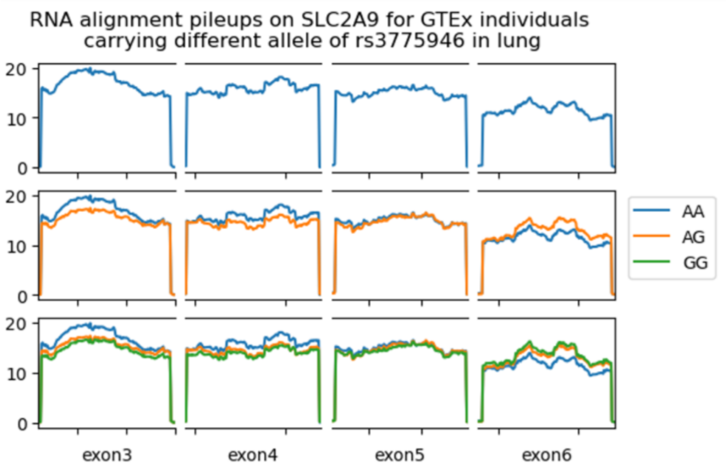

The meta-analysis signal colocalized with a *SLC2A9* sQTL. The RNA pileup plot shows the aligned reads in the indicated GTEx tissue for the indicated exons that were included in the LeafCutter splicing cluster. Unlike an eQTL, a subset of exons show differences in the amount of reads aligned when stratified by the indicated genotype, supporting that this is a sQTL. *SLC2A9* was also the proximal gene, therefore we concluded that *SLC2A9* was the best supported putative causal gene.

rs4691895 (chr4:163127047:G:C)

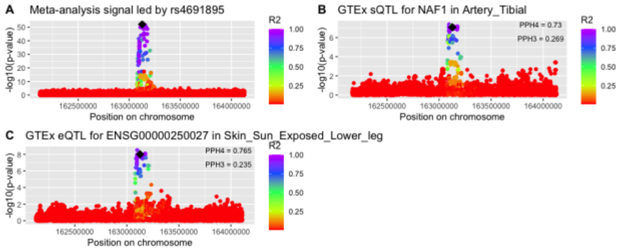

	rsid	hgnc_symbol	dataset	tissue	PPH3	PPH4
1	rs4691895	NAF1	GTEx_eQTL	Thyroid	0.3741141	0.6099393
2	rs4691895	NAF1	GTEx_sQTL	Artery_Tibial	0.2692093	0.7300762
3	rs4691895	ENSG00000250027	GTEx_eQTL	Skin_Sun_Exposed_Lower_leg	0.2347356	0.7651807
4	rs4691895	ENSG00000250027	DICE	TH2	0.4014418	0.5168376

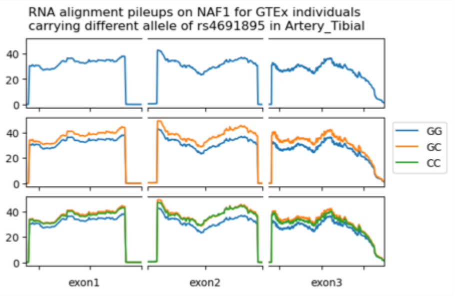

The only characterized gene QTL this meta-analysis signal colocalized with was a *NAF1* sQTL. *NAF1* is also a known telomere regulation gene and the gene proximal to the signal. The RNA pileup plot shows the aligned reads in the indicated GTEx tissue for the indicated exons that were included in the LeafCutter splicing cluster. Unlike an eQTL, a subset of exons show differences in the amount of reads aligned when stratified by the indicated genotype, supporting that this is a sQTL.Therefore, we concluded that *NAF1* was the best supported putative causal gene.

rs3131064 (chr6:30796116:T:C)

	rsid	hgnc_symbol	dataset	tissue	PPH3	PPH4
1	rs3131064	TNXB	eQTLGen	Whole_Blood	0.005108423	0.9948916
2	rs3131064	CSNK2B	eQTLGen	Whole_Blood	0.288034393	0.7119656
3	rs3131064	POU5F1	GTEx_eQTL	Thyroid	0.109280002	0.8907200
4	rs3131064	CCHCR1	GTEx_eQTL	Heart_Left_Ventricle	0.268112750	0.7318872
5	rs3131064	EGFL8	eQTLGen	Whole_Blood	0.192744415	0.8049338

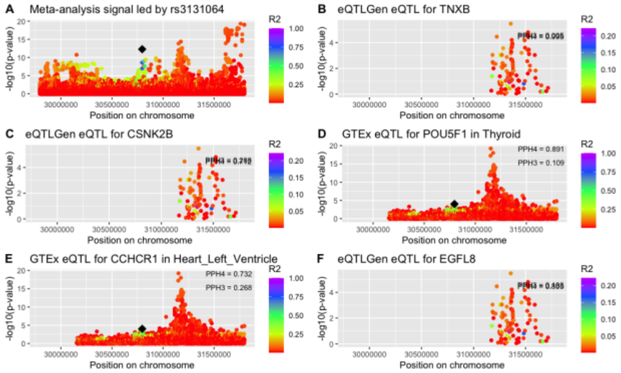

This meta-analysis signal is near the *HLA* locus and there may be several independent signals within this region. We defined signals based on position alone, therefore we are treating this region as a single signal. rs3131064 was considered the lead SNP because lead SNPs were chosen by ordering the genome-wide significant SNPs by p-value, selecting the top SNP within a 1 megabase region, and then removing any other SNPs within 1 megabase of that top SNP. The peaks adjacent to rs3131064 within panel A are within 1 megabase of rs1150748 and were therefore excluded from being considered the lead SNP for this signal. One of these adjacent signals, led by rs1265156, colocalized with with QTLs for *POU5F1* and *CCHCR1*. The proximal gene was *HCG20*. We concluded that *POU5F1* was the best supported putative causal gene for this region.

rs1150748 (chr6:31804139:G:C)

	rsid	hgnc_symbol	dataset	tissue	PPH3	PPH4
1	rs1150748	VARS2	GTEx_eQTL	Pancreas	0.1608101	0.8391402
2	rs1150748	MUCL3	GTEx_eQTL	Nerve_Tibial	0.3258736	0.6155796
3	rs1150748	HLA-DQA1	GTEx_sQTL	Pancreas	0.2755417	0.7178696
4	rs1150748	EHMT2	GTEx_eQTL	Testis	0.2709409	0.7261355
5	rs1150748	BAG6	GTEx_eQTL	Brain_Hippocampus	0.0864677	0.9134108
6	rs1150748	BAG6	GTEx_sQTL	Thyroid	0.2439565	0.7544198
7	rs1150748	AIF1	GTEx_sQTL	Adipose_Subcutaneous	0.3509177	0.6490804
8	rs1150748	PPT2	eQTLGen	Whole_Blood	0.1062758	0.8937242
9	rs1150748	HLA-DQA2	GTEx_eQTL	Artery_Aorta	0.3070131	0.6926297
10	rs1150748	HLA-DQA2	GTEx_sQTL	Pancreas	0.2666779	0.7269180

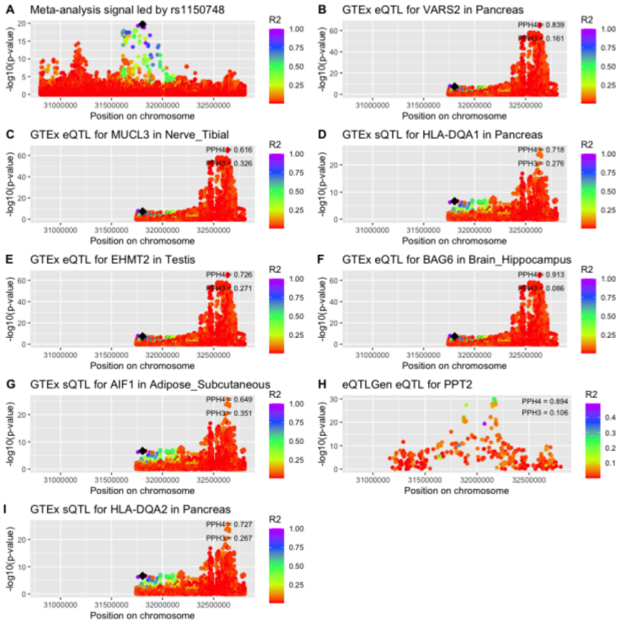

This meta-analysis signal is near the *HLA* locus and there may be several independent signals within this region. We defined signals based on position alone, therefore we are treating this region as a single signal. rs1150748 was considered the lead SNP because lead SNPs were chosen by ordering the genome-wide significant SNPs by p-value, selecting the top SNP within a 1 megabase region, and then removing any other SNPs within 1 megabase of that top SNP. This signal colocalized well with several gene QTLs but the association signal structure was best captured by the *BAG6* QTL. *LSM2* was the proximal gene. We concluded that *BAG6* was the best supported putative causal gene.

rs6968500 (chr7:124791668:G:C)

	rsid	hgnc_symbol	dataset	tissue	PPH3	PPH4
1	rs6968500	POT1-AS1	GTEx_eQTL	Thyroid	0.06175501	0.9382314

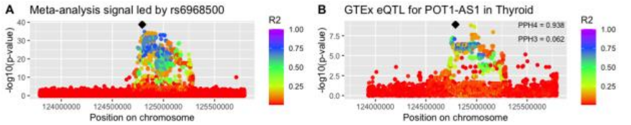

This meta-analysis signal colocalized with a QTL for *POT1-AS1*. The proximal gene was *C7orf77*. *POT1*, 30 kilobases away from the lead SNP, has known roles in telomere length regulation. Therefore, we concluded that *POT1* was the most likely putative causal gene.

rs10954213 (chr7:128949373:A:G)

	rsid	hgnc_symbol	dataset	tissue	PPH3	PPH4
1	rs10954213	TNPO3	GTEx_eQTL	Brain_Putamen_basal_ganglia	0.42356764	0.5696414
2	rs10954213	IRF5	GTEx_eQTL	Testis	0.19661305	0.7978561
3	rs10954213	ENSG00000275106	GTEx_eQTL	Spleen	0.08300514	0.9169949

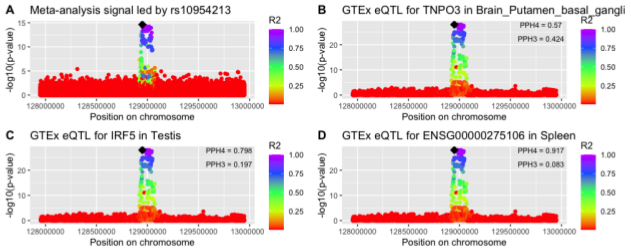

This meta-analysis signal had the strongest colocalization with *IRF5* QTLs, which is also the proximal gene. In addition, TWAS analysis suggested that *IRF5* was the underlying causal gene for this signal. Therefore, we concluded that *IRF5* was the best supported putative causal gene.

rs10958468 (chr8:55749589:G:A)

	rsid	hgnc_symbol	dataset	tissue	PPH3	PPH4
1	rs10958468	TGS1	GTEx_eQTL	Skin_Sun_Exposed_Lower_leg	0.3122899	0.6876307
2	rs10958468	TGS1	eQTLGen	Whole_Blood	0.4804886	0.5193802
3	rs10958468	TMEM68	GTEx_eQTL	Esophagus_Gastroesophageal_Junction	0.4799957	0.5193636

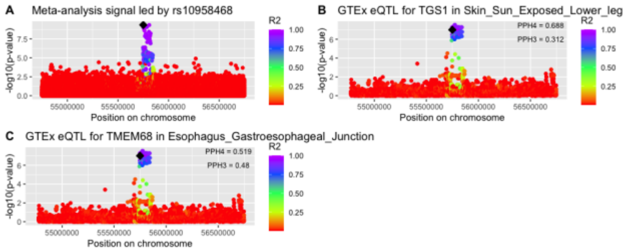

This meta-analysis signal only suggestively colocalized with QTLs. The proximal gene was *TMEM68*. In the absence of strong colocalization results, we conclude that *TMEM68* is the most likely putative causal gene.

rs10111287 (chr8:94566198:C:T)

	rsid	hgnc_symbol	dataset	tissue	PPH3	PPH4
1	rs10111287	VIRMA	GTEx_eQTL	Thyroid	0.07729804	0.9225971
2	rs10111287	VIRMA	GTEx_sQTL	Lung	0.36459207	0.6337992
3	rs10111287	VIRMA	eQTLGen	Whole_Blood	0.07057065	0.9274511
4	rs10111287	VIRMA-DT	GTEx_eQTL	Colon_Sigmoid	0.45165026	0.5424639
5	rs10111287	VIRMA-DT	eQTLGen	Whole_Blood	0.02614059	0.9729704

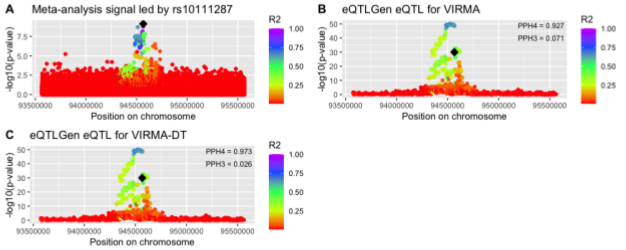

This meta-analysis signal best colocalized with *VIRMA* QTLs. *VIRMA* is also the proximal gene. Therefore, we concluded that *VIRMA* is the best supported putative causal gene.

rs62560860 (chr9:34077464:G:A)

	rsid	hgnc_symbol	dataset	tissue	PPH3	PPH4
1	rs62560860	IL11RA	GTEx_sQTL	Adipose_Subcutaneous	0.08446549	0.915153

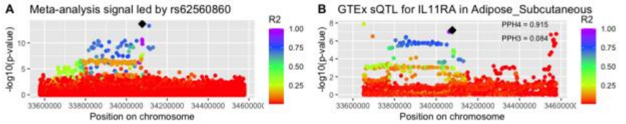

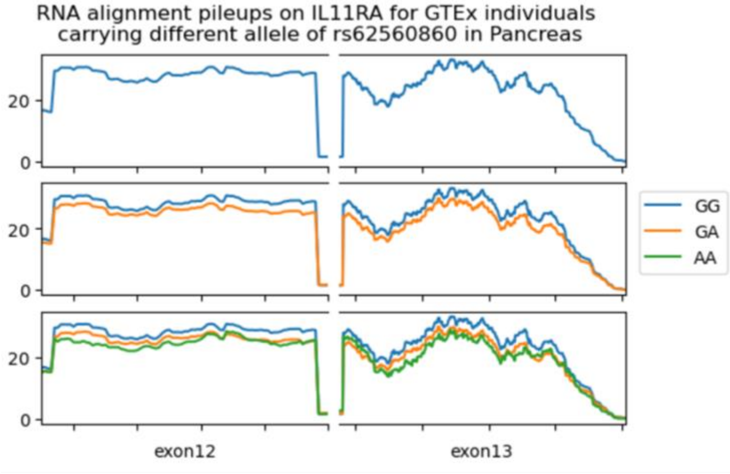

This meta-analysis signal only colocalized with an *IL11RA* sQTL. The proximal gene was *DCAF12*. Note that plot is 1 megabase wide instead of 2 megabases wide to improve visualization of the sQTL because there is a nearby SNP, rs11575580, that has a strong association ($p=3.04 \times 10^{-119}$) but r^2 with meta-analysis lead SNP = 0.0169 and did not contribute to the colocalization signal. To improve clarity, we reduced the plot region to 1 megabase centered on the meta-analysis lead SNP. The RNA pileup plot shows the aligned reads in the indicated GTEx tissue for the indicated exons that were included in the LeafCutter splicing cluster. Unlike an eQTL, a subset of exons show differences in the amount of reads aligned when stratified by the indicated genotype, supporting that this is a sQTL.Given the colocalization analysis results we concluded that *ILR11A* was the most supported putative causal gene.

rs7923385 (chr10:97357340:A:T)

	rsid	hgnc_symbol	dataset	tissue	PPH3	PPH4
1	rs7923385	RRP12	GTEx_eQTL	Brain_Frontal_Cortex_BA9	0.3655131	0.6215017
2	rs7923385	ENSG00000225850	GTEx_eQTL	Whole_Blood	0.3591939	0.5768156

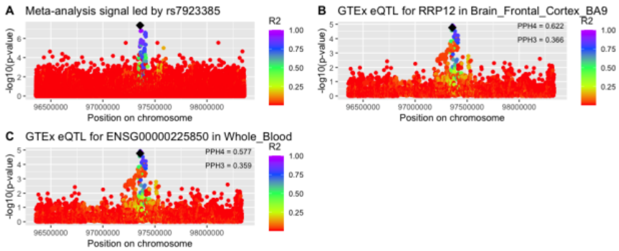

TWAS nominated *RRP12* which is also the proximal gene. In addition, there was suggestive colocalization analysis results with an *RRP12* eQTL. Therefore, we concluded that *RRP12* was the most supported putative causal gene.

rs11190126 (chr10:99512032:A:C)

	rsid	hgnc_symbol	dataset	tissue	PPH3	PPH4
1	rs11190126	LINC01475	GTEx_sQTL	Spleen	0.4368107	0.5616428

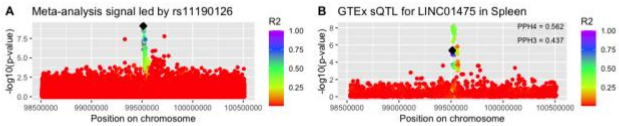

There were no strong colocalization results for this meta-analysis signal (PPH4 > 0.7). The proximal coding gene was *NKX2-3*, therefore we concluded that *NKX2-3* is the most likely putative causal gene.

rs2475215 (chr10:103900944:T:C)

	rsid	hgnc_symbol	dataset	tissue	PPH3	PPH4
1	rs2475215	SLK	GTEx_eQTL	Artery_Aorta	0.0768511	0.9229903
2	rs2475215	STN1	GTEx_eQTL	Brain_Putamen_basal_ganglia	0.2820507	0.6415053

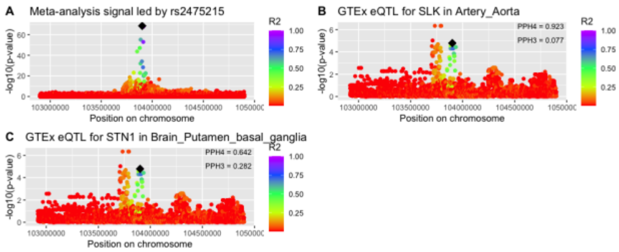

This meta-analysis signal best colocalized with an *SLK* QTL. However, *OBFC1* (aka *STN1*) is a known telomere length regulation gene located 17 kilobases away and an *OBFC1* eQTL had suggestive colocalization with this meta-analysis signal. Given the known biology, we concluded that *OBFC1* was the most likely putative causal signal.

rs582297 (chr11:108294680:C:G)

	rsid	hgnc_symbol	dataset	tissue	PPH3	PPH4
1	rs582297	NPAT	DICE	CD8_NAIVE	0.4180164	0.5819775
2	rs582297	ATM	GTEx_eQTL	Esophagus_Muscularis	0.2924375	0.7075625
3	rs582297	ATM	GTEx_sQTL	Lung	0.4787983	0.5201501

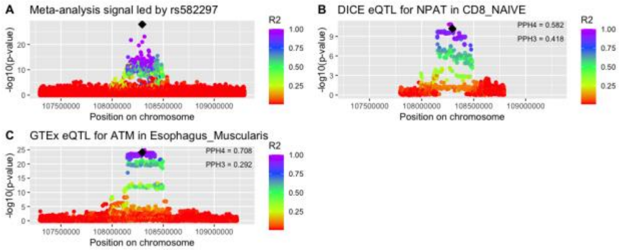

This meta-analysis signal colocalized with QTLs for *ATM*. TWAS analysis supported *NPAT* as the likely causal gene. *ATM* was the proximal gene and has known roles in telomere length regulation. Therefore, we concluded that *ATM* was the best supported putative causal gene.

rs74892322 (chr12:120533371:A:T)

	rsid	hgnc_symbol	dataset	tissue	PPH3	PPH4
1	rs74892322	COX6A1	GTEx_eQTL	Pituitary	0.3706755	0.6217557
2	rs74892322	SRSF9	GTEx_eQTL	Testis	0.3678655	0.5345490
3	rs74892322	POP5	GTEx_eQTL	Brain_Nucleus_accumbens_basal_ganglia	0.3512162	0.5657644
4	rs74892322	GATC	GTEx_eQTL	Nerve_Tibial	0.1452442	0.8538557

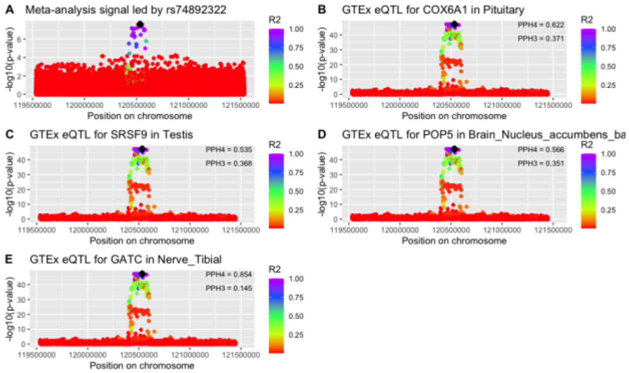

This meta-analysis signal suggestively or strongly colocalized with QTLs for *COX6A1*, *SRSF9*, *POP5*, and *GATC.* The proximal gene was *RNF10*. Given our results from Figures 6 and 7, we concluded that the best supported putative causal gene was *POP5*.

rs28755851 (chr12:123001735:A:T)

	rsid	hgnc_symbol	dataset	tissue	PPH3	PPH4
1	rs28755851	ABCB9	GTEx_eQTL	Skin_Not_Sun_Exposed_Suprapubic	0.2760382	0.7221415
2	rs28755851	ABCB9	DICE	TREG_NAIVE	0.4530929	0.5408847
3	rs28755851	ARL6IP4	GTEx_eQTL	Stomach	0.4052089	0.5921491

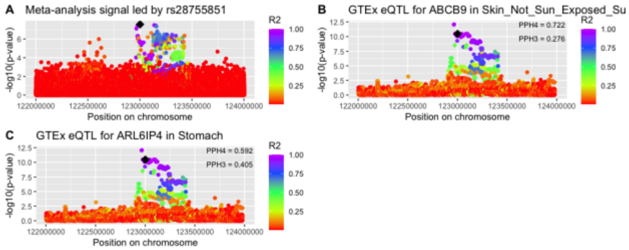

This meta-analysis signal colocalized with an *ABCB9* eQTL in GTEx and this was replicated in DICE. TWAS analysis supported *MPHOSPH9*. The proximal gene was *PITPNM2*. However, *ZCCHC8* has known roles in telomere length regulation and is 530 kilobases away. We concluded that *ZCCHC8* was the most likely putative causal gene despite lack of colocalization.

rs1411041 (chr13:41150640:A:T)

	rsid	hgnc_symbol	dataset	tissue	PPH3	PPH4
1	rs1411041	KBTBD7	GTEx_eQTL	Whole_Blood	0.06054376	0.9394562
2	rs1411041	KBTBD7	DICE	MONOCYTES	0.14224342	0.6394897
3	rs1411041	KBTBD6	GTEx_eQTL	Spleen	0.07735818	0.9078444

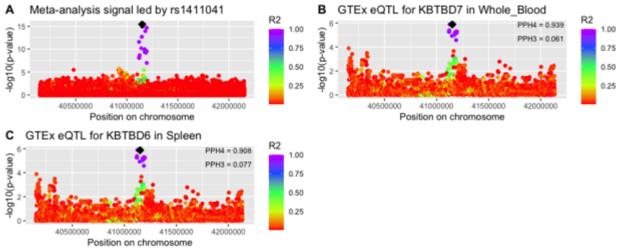

This meta-analysis signal colocalized well with *KBTBD6* and *KBTBD7* QTLs. TWAS analysis supported *KBTBD7* as the underlying causal gene. *KBTBD6* is the proximal gene (23 kilobases from the lead SNP whereas *KBTBD7* is 39 kilobases). Particularly considering our validation experiments, we are unable to choose a single putative causal gene for this signal. We label the Manhattan plot in Figure 1 *KBTBD6* for clarity.

rs4902358 (chr14:65075759:A:G)

	rsid	hgnc_symbol	dataset	tissue	PPH3	PPH4
1	rs4902358	MAX	GTEx_sQTL	Esophagus_Muscularis	0.1808947	0.8177149
2	rs4902358	MAX	eQTLGen	Whole_Blood	0.3698076	0.6273319
3	rs4902358	ENSG00000272158	GTEx_eQTL	Breast_Mammary_Tissue	0.3065125	0.5927292

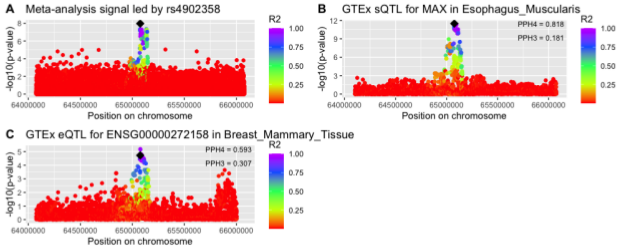

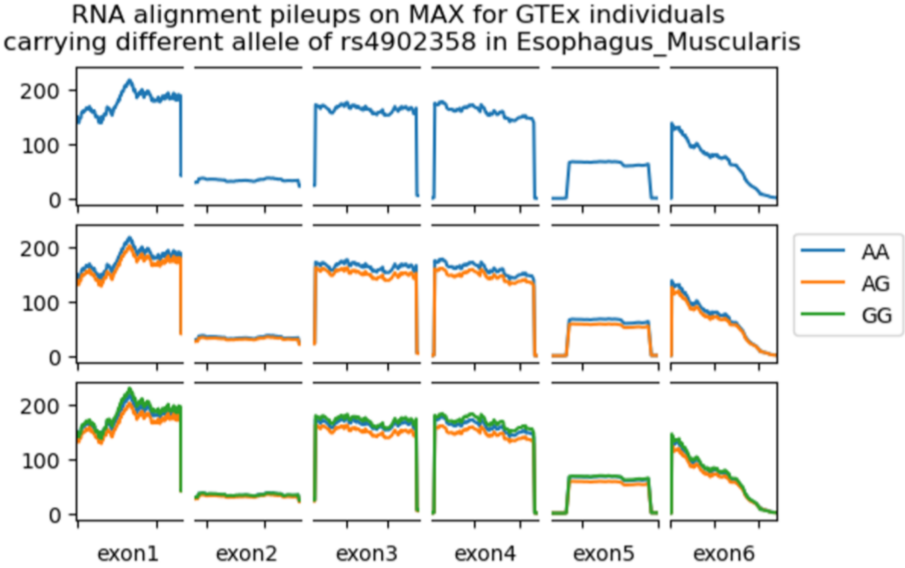

This meta-analysis signal best colocalized with *MAX* QTLs. *MAX* is also the proximal gene. The RNA pileup plot shows the aligned reads in the indicated GTEx tissue for the indicated exons that were included in the LeafCutter splicing cluster. Unlike an eQTL, a subset of exons show differences in the amount of reads aligned when stratified by the indicated genotype, supporting that this is a sQTL.Therefore, we concluded that *MAX* was the best supported putative causal gene.

rs11623533 (chr14:91446739:C:A)

	rsid	hgnc_symbol	dataset	tissue	PPH3	PPH4
1	rs11623533	CCDC88C	GTEx_eQTL	Brain_Frontal_Cortex_BA9	0.287627	0.650732

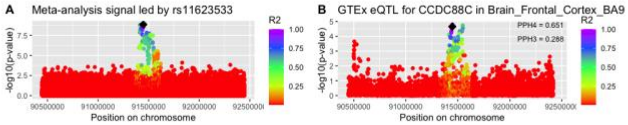

This meta-analysis signal did not strongly colocalize with any QTLs. The lead SNP for this signal intergenic, 10.8 kilobases downstream *PPP4R3A* and 28.9 kilobases upstream of *CCDC88C*. In the absence of strong colocalization results, we concluded that *PPP4R3A* is the most likely causal gene.

rs2887399 (chr14:95714358:G:T)

	rsid	hgnc_symbol	dataset	tissue	PPH3	PPH4
1	rs2887399	TCL1A	GTEx_eQTL	Whole_Blood	0.2549033	0.7138769

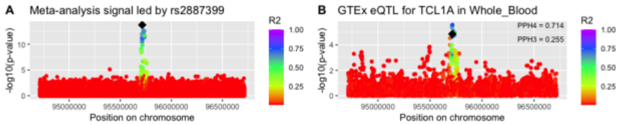

This meta-analysis signal best colocalized with *TCL1A* QTLs. TWAS supported *TCL1A* as associated with telomere length. *TCL1A* was also the proximal gene. Therefore, we concluded that *TCL1A* was the best supported putative casual gene.

rs113119217 (chr15:50073451:T:A)

	rsid	hgnc_symbol	dataset	tissue	PPH3	PPH4
1	rs113119217	ATP8B4	GTEx_eQTL	Colon_Transverse	0.1466633	0.8002314
2	rs113119217	ATP8B4	DICE	MONOCYTES	0.1353581	0.5583945
3	rs113119217	ATP8B4	eQTLGen	Whole_Blood	0.2677138	0.7322862

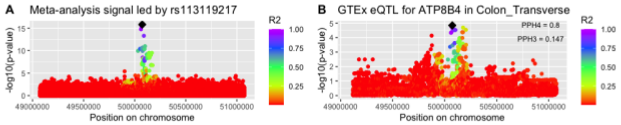

This meta-analysis signal best colocalized with QTLs for *ATP8B4* in multiple QTL datasets. *ATP8B4* is also the proximal gene. In addition, TWAS analysis supported *ATP8B4* as the likely causal gene underlying this signal. Therefore, we concluded that *ATP8B4* was the best supported putative causal gene.

rs12934863 (chr16:68043168:A:G)

	rsid	hgnc_symbol	dataset	tissue	PPH3	PPH4
1	rs12934863	NFATC3	GTEx_eQTL	Adipose_Visceral_Omentum	0.05336006	0.9448775
2	rs12934863	CTCF	GTEx_eQTL	Adipose_Subcutaneous	0.19070371	0.6361457
3	rs12934863	SLC7A6	GTEx_eQTL	Cells_Cultured_fibroblasts	0.16263505	0.8324301
4	rs12934863	ESRP2	eQTLGen	Whole_Blood	0.08158831	0.9158031
5	rs12934863	PRMT7	GTEx_eQTL	Brain_Anterior_cingulate_cortex_BA24	0.12906491	0.8664440
6	rs12934863	SLC9A5	GTEx_eQTL	Adipose_Subcutaneous	0.16762805	0.6959520
7	rs12934863	DPEP2	eQTLGen	Whole_Blood	0.03672786	0.9619414
8	rs12934863	DUS2	GTEx_eQTL	Cells_Cultured_fibroblasts	0.03939997	0.9592253
9	rs12934863	DUS2	GTEx_sQTL	Skin_Sun_Exposed_Lower_leg	0.12631617	0.8433910
10	rs12934863	DUS2	eQTLGen	Whole_Blood	0.44696862	0.5400439
11	rs12934863	KCTD19	GTEx_eQTL	Testis	0.32206306	0.6631488
12	rs12934863	B3GNT9	GTEx_eQTL	Artery_Tibial	0.35993685	0.6242723
13	rs12934863	ENSG00000263276	GTEx_eQTL	Lung	0.17583320	0.6016294
14	rs12934863	ENSG00000279649	GTEx_eQTL	Cells_Cultured_fibroblasts	0.21883581	0.5968948

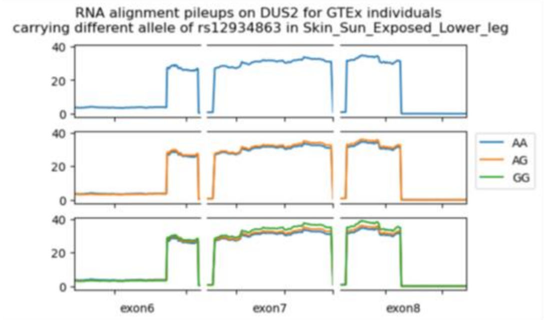

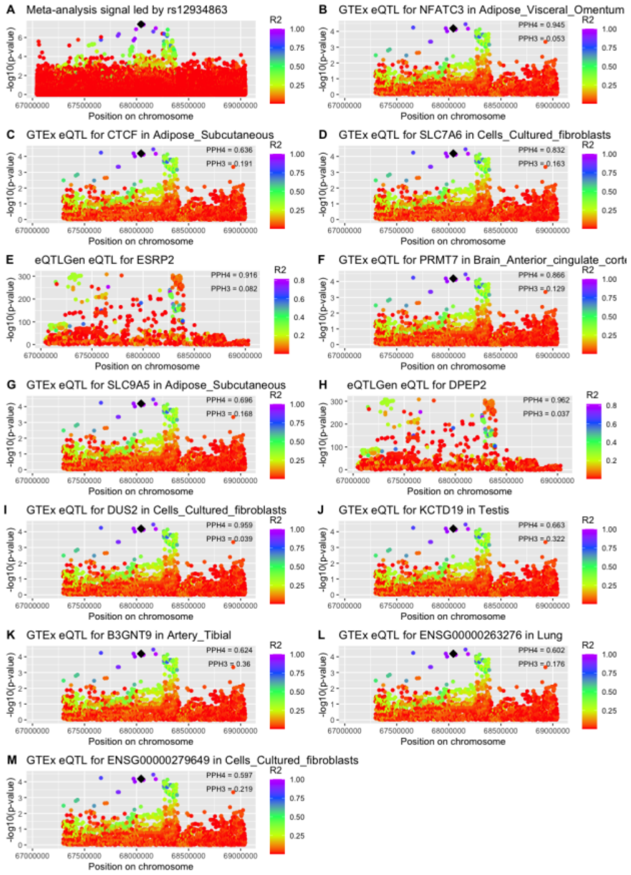

This meta-analysis signal colocalized strongly with QTLs for *NFATC3, SLC7A6, ESRP2, PRMT7, DPEP2,* and *DUS2.*The proximal gene was *DUS2*. The RNA pileup plot shows the aligned reads in the indicated GTEx tissue for the indicated exons that were included in the LeafCutter splicing cluster. Unlike an eQTL, a subset of exons show differences in the amount of reads aligned when stratified by the indicated genotype, supporting that this is a sQTL. We concluded that the best supported putative causal gene was *DUS2*.

rs9939870 (chr16:69362682:C:T)

	rsid	hgnc_symbol	dataset	tissue	PPH3	PPH4
1	rs9939870	NIP7	GTEx_eQTL	Thyroid	0.1835250	0.8164750
2	rs9939870	TERF2	GTEx_eQTL	Esophagus_Mucosa	0.1899055	0.8100943
3	rs9939870	TERF2	eQTLGen	Whole_Blood	0.0175697	0.9824303
4	rs9939870	VPS4A	GTEx_eQTL	Muscle_Skeletal	0.2834250	0.5375818
5	rs9939870	VPS4A	GTEx_sQTL	Testis	0.4661460	0.5306913
6	rs9939870	COG8	GTEx_eQTL	Thyroid	0.3069324	0.6170980
7	rs9939870	COG8	GTEx_sQTL	Adipose_Subcutaneous	0.1877379	0.8122532
8	rs9939870	PDF	GTEx_eQTL	Small_Intestine_Terminal_Ileum	0.3156775	0.6839057
9	rs9939870	PDF	GTEx_sQTL	Adipose_Subcutaneous	0.1877379	0.8122532

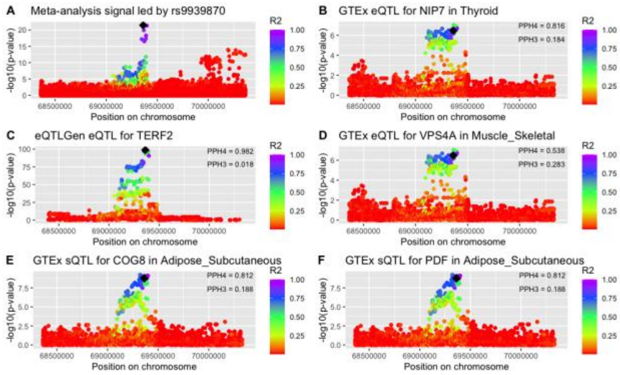

This meta-analysis signal colocalized strongly with QTLs for *NIP7*, *TERF2*, *COG8*, and *PDF*. TWAS suggested that *TERF2* is associated with telomere length. The proximal gene was *TERF2* and *TERF2* has known roles in telomere length regulation. Therefore, we concluded that *TERF2* is the best supported putative causal gene.

rs12149396 (chr16:70392835:A:C)

	rsid	hgnc_symbol	dataset	tissue	PPH3	PPH4
1	rs12149396	PDPR	GTEx_eQTL	Esophagus_Muscularis	0.35375494	0.6433592
2	rs12149396	AARS1	GTEx_eQTL	Muscle_Skeletal	0.27673851	0.7232615
3	rs12149396	CLEC18A	GTEx_eQTL	Brain_Substantia_nigra	0.40534487	0.5946551
4	rs12149396	CLEC18C	GTEx_eQTL	Adrenal_Gland	0.02618274	0.9738173
5	rs12149396	ENSG00000247228	GTEx_eQTL	Testis	0.02593613	0.9740639

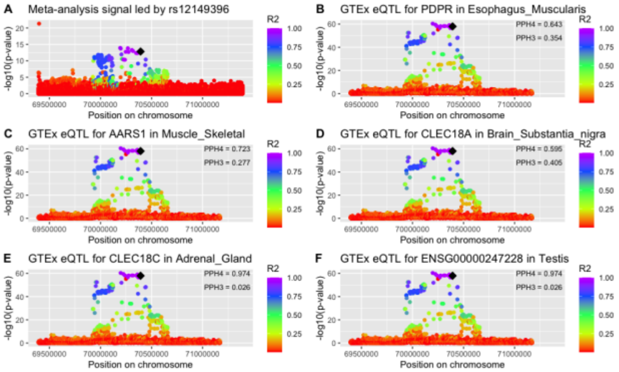

This meta-analysis signal strongly colocalized with *AARS1* and *CLEC18C* QTLs. The proximal gene was *ST3GAL2*. Based on the strength of colocalization, we concluded that *CLEC18C* was the best supported putative causal gene.

rs7193541 (chr16:74630845:T:C)

	rsid	hgnc_symbol	dataset	tissue	PPH3	PPH4
1	rs7193541	RFWD3	GTEx_eQTL	Muscle_Skeletal	0.0061681692	0.9938318
2	rs7193541	RFWD3	GTEx_sQTL	Cells_Cultured_fibroblasts	0.0003784313	0.9996216
3	rs7193541	RFWD3	DICE	TFH	0.1124013390	0.5893663

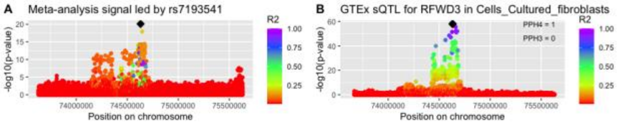

This meta-analysis signal best colocalized with *RFWD3* QTLs. *RFWD3* was also the proximal gene. We note that LeafCutter visualization of the splicing pattern supported an effect of the lead SNP at the association signal over different *RFWD3* splicing patterns as discussed in greater detail in the main text. Based on these results, we concluded that *RFWD3* was the best supported putative causal gene.

rs6564996 (chr16:82173937:T:C)

	rsid	hgnc_symbol	dataset	tissue	PPH3	PPH4
1	rs6564996	MPHOSPH6	GTEx_eQTL	Stomach	0.02675088	0.9732491
2	rs6564996	MPHOSPH6	GTEx_sQTL	Whole_Blood	0.18700181	0.8116272

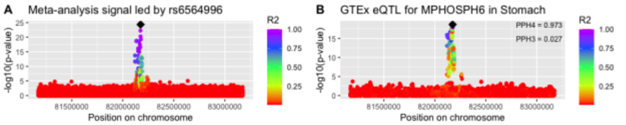

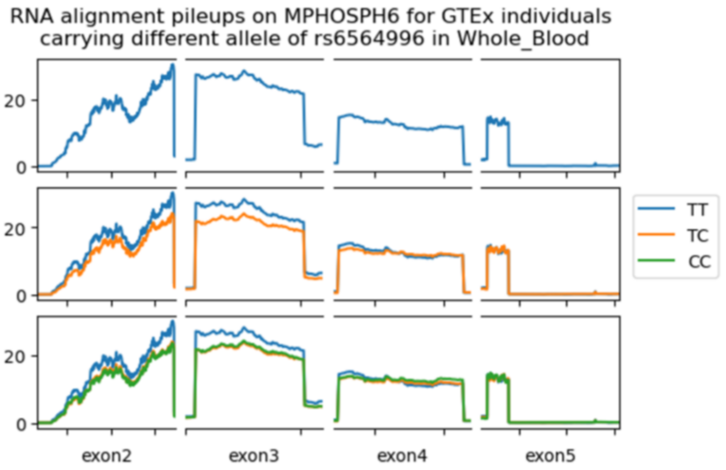

This meta-analysis signal best colocalized with *MPHOSPH6* QTLs. *MPHOSPH6* was also the proximal gene. The RNA pileup plot shows the aligned reads in the indicated GTEx tissue for the indicated exons that were included in the LeafCutter splicing cluster. Unlike an eQTL, a subset of exons show differences in the amount of reads aligned when stratified by the indicated genotype, supporting that this is a sQTL. Therefore, we concluded that *MPHOSPH6* was the best supported putative causal gene.

rs59922886 (chr17:8236454:A:T)

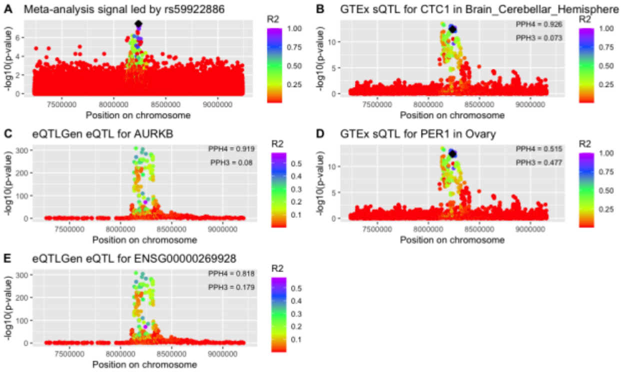

	rsid	hgnc_symbol	dataset	tissue	PPH3	PPH4
1	rs59922886	CTC1	GTEx_eQTL	Skin_Sun_Exposed_Lower_leg	0.13638793	0.8611165
2	rs59922886	CTC1	GTEx_sQTL	Brain_Cerebellar_Hemisphere	0.07278529	0.9258539
3	rs59922886	CTC1	DICE	M2	0.32645754	0.6408274
4	rs59922886	AURKB	eQTLGen	Whole_Blood	0.07964168	0.9188518
5	rs59922886	PER1	GTEx_sQTL	Ovary	0.47688311	0.5151215
6	rs59922886	ENSG00000269928	DICE	MONOCYTES	0.18350092	0.8118216
7	rs59922886	ENSG00000269928	eQTLGen	Whole_Blood	0.17924724	0.8175740

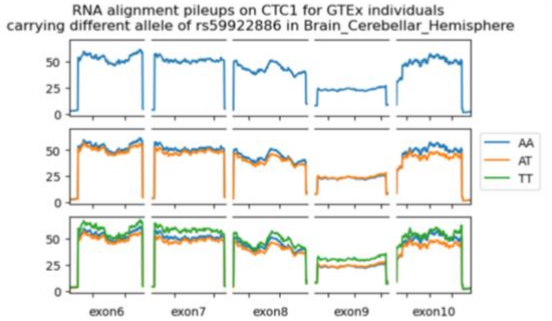

This meta-analysis signal strongly colocalized with QTLs for *CTC1* and *AURKB*. *CTC1* is the proximal gene and has known roles in telomere length regulation. The RNA pileup plot shows the aligned reads in the indicated GTEx tissue for the indicated exons that were included in the LeafCutter splicing cluster. Unlike an eQTL, a subset of exons show differences in the amount

of reads aligned when stratified by the indicated genotype, supporting that this is a sQTL. Therefore, we concluded that *CTC1* was the best supported putative causal gene.

rs208011 (chr17:48180983:A:G)

	rsid	hgnc_symbol	dataset	tissue	PPH3	PPH4
1	rs208011	NFE2L1	GTEx_eQTL	Thyroid	0.3156817	0.6838702
2	rs208011	CBX1	GTEx_eQTL	Colon_Transverse	0.3151132	0.6844636

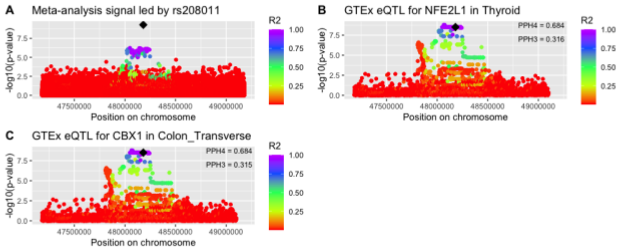

This meta-analysis signal did not strongly colocalize with any QTLs. The proximal gene was *SKAP1*. We note that *CBX1* was one of the genes chosen for experimental validation and while overexpression of *CBX1* increased telomere length (Supplementary Figure 7A). Together with the suggestive colocalization data, we conclude that *CBX1* is the best supported putative causal gene.

rs144204502 (chr17:78187152:C:T)

	rsid	hgnc_symbol	dataset	tissue	PPH3	PPH4
1	rs144204502	TK1	GTEx_eQTL	Brain_Nucleus_accumbens_basal_ganglia	0.0003874088	0.9996031

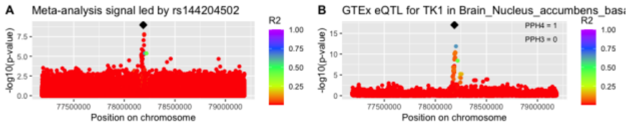

This meta-analysis signal best colocalized with *TK1* QTLs. *TK1* was also the proximal gene. Therefore, we concluded that *TK1* is the best supported putative casual gene.

rs2124616 (chr18:661917:G:A)

	rsid	hgnc_symbol	dataset	tissue	PPH3	PPH4
1	rs2124616	TYMSOS	GTEx_eQTL	Esophagus_Mucosa	0.08837448	0.9115152
2	rs2124616	ENSG00000264635	GTEx_eQTL	Breast_Mammary_Tissue	0.28491231	0.7041308

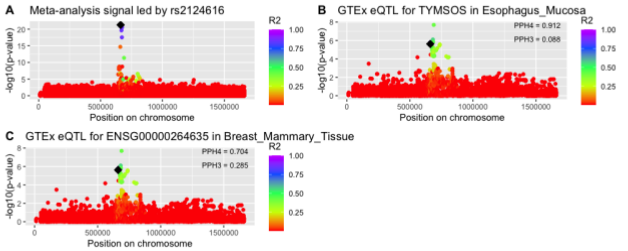

This meta-analysis signal best colocalized with a *TYMSOS* QTL. *TYMS* was the proximal gene. We concluded that *TYMSOS* was the best supported putative causal gene.

rs139955496 (chr18:54306914:A:G)

	rsid	hgnc_symbol	dataset	tissue	PPH3	PPH4
1	rs139955496	POLI	GTEx_sQTL	Nerve_Tibial	0.420734	0.5783875

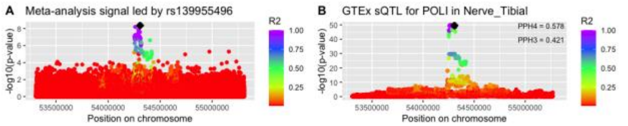

This meta-analysis suggestively colocalized with a *POLI* sQTL and *POLI* is the proximal gene. Therefore, we concluded that *POLI* is the best supported putative causal gene.

rs8105767 (chr19:22032639:A:G)

	rsid	hgnc_symbol	dataset	tissue	PPH3	PPH4
1	rs8105767	ZNF208	GTEx_sQTL	Pituitary	8.266567e-02	0.9162672
2	rs8105767	ZNF257	GTEx_eQTL	Brain_Hypothalamus	7.387153e-04	0.9992613
3	rs8105767	ZNF257	DICE	TREG_MEM	3.982662e-01	0.5992816
4	rs8105767	ZNF257	eQTLGen	Whole_Blood	4.313417e-05	0.9999569

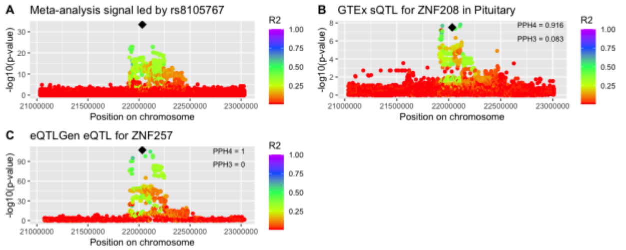

This meta-analysis signal colocalized with *ZNF257* and *ZNF208* QTLs. The colocalization with *ZNF257* QTLs was replicated in multiple datasets. *ZNF257* was also the proximal gene. Therefore, we concluded that *ZNF257* was the best supported putative causal gene.

rs79476302 (chr20:36967509:T:A)

	rsid	hgnc_symbol	dataset	tissue	PPH3	PPH4
1	rs79476302	RBL1	GTEx_eQTL	Cells_Cultured_fibroblasts	0.405355	0.5940007

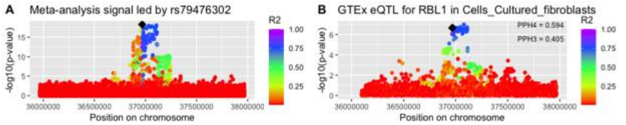

This meta-analysis signal suggestively colocalized with *RBL1* QTLs. *SAMHD1* was the proximal signal. Therefore, we concluded that *SAMHD1* was the best supported putative causal gene.

rs114703330 (chr20:63678039:T:C)

	rsid	hgnc_symbol	dataset	tissue	PPH3	PPH4
1	rs114703330	SLC2A4RG	GTEx_eQTL	Cells_Cultured_fibroblasts	0.03660651	0.9604923
2	rs114703330	STMN3	GTEx_eQTL	Artery_Aorta	0.10977515	0.8901090
3	rs114703330	TNFRSF6B	GTEx_eQTL	Adipose_Visceral_Omentum	0.22731210	0.6412560
4	rs114703330	RTEL1	GTEx_eQTL	Heart_Atrial_Appendage	0.08571417	0.9134698

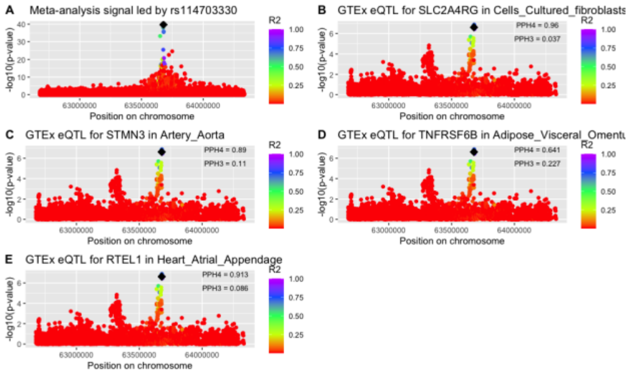

This meta-analysis signal strongly colocalized with QTLs for *SLC2A4RG*, *STMN3*, and *RTEL1*. TWAS analysis supported *LIME1* as a putative causal gene for this meta-analysis signal. The proximal gene was *RTEL1* and *RTEL1* has known roles in telomere length regulation. Therefore, we concluded that *RTEL1* was the best supported putative causal gene.

rs28663120 (chr22:16973188:T:C)

	rsid	hgnc_symbol	dataset	tissue	PPH3	PPH4
1	rs28663120	GAB4	DICE	CD8_NAIVE	0.2605901	0.7326439

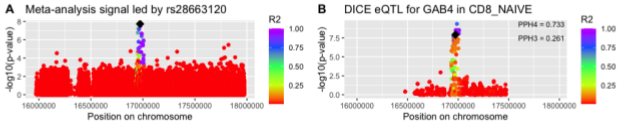

This meta-analysis signal colocalized with QTLs for *GAB4*. *GAB4* was the proximal gene. Therefore, we concluded that *GAB4* is the best supported putative causal gene.

rs131784 (chr22:50543007:G:A)

	rsid	hgnc_symbol	dataset	tissue	PPH3	PPH4
1	rs131784	TYMP	GTEx_eQTL	Pancreas	0.04245866	0.9574469
2	rs131784	TYMP	GTEx_sQTL	Adipose_Visceral_Omentum	0.10697766	0.8930188
3	rs131784	TYMP	eQTLGen	Whole_Blood	0.39804704	0.6019395
4	rs131784	LMF2	GTEx_eQTL	Brain_Nucleus_accumbens_basal_ganglia	0.33594449	0.6402745
5	rs131784	ENSG00000130489	GTEx_eQTL	Whole_Blood	0.09454167	0.9054552
6	rs131784	ENSG00000130489	eQTLGen	Whole_Blood	0.19005293	0.8099405
7	rs131784	ODF3B	GTEx_eQTL	Colon_Transverse	0.07377038	0.9191106

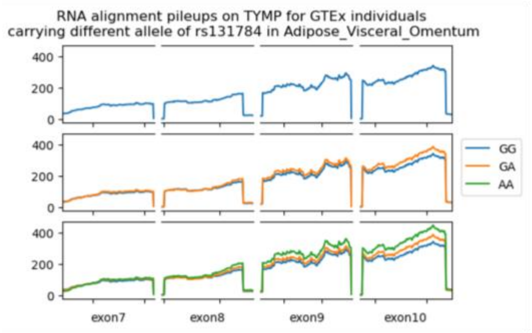

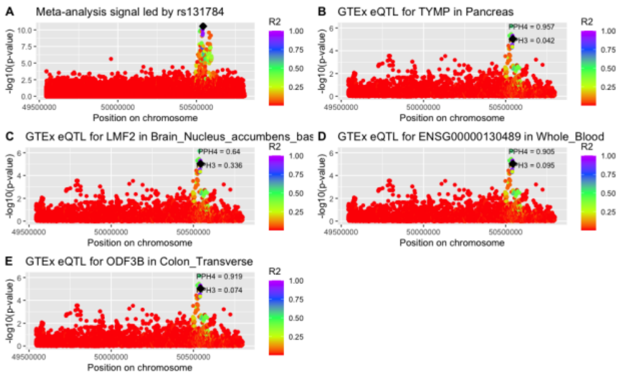

This meta-analysis signal colocalized best with QTLs for *TYMP*, and *ODF3B*. The proximal gene was *KLHDC7B*. TWAS analysis supported both *TYMP* and *SCO2*. The RNA pileup plot shows the aligned reads in the indicated GTEx tissue for the indicated exons that were included in the LeafCutter splicing cluster. Unlike an eQTL, a subset of exons show differences in the amount of reads aligned when stratified by the indicated genotype, supporting that this is a sQTL. As colocalization was strongest with *TYMP* QTLs, we concluded that *TYMP* was the best supported putative causal gene.

rs12394264 (chrX:66015290:G:A)

	rsid	hgnc_symbol	dataset	tissue	PPH3	PPH4
1	rs12394264	MIR223HG	GTEx_eQTL	Esophagus_Muscularis	0.05514321	0.9448568

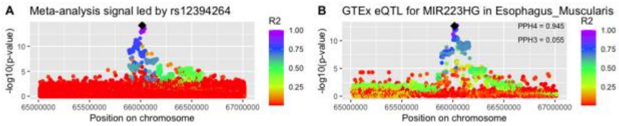

This meta-analysis signal best colocalized with QTLs for *MIR223HG*. *MIR223HG* was also the proximal gene. Therefore, we concluded that the best supported putative casual gene was *MIR223HG*.

rs5945232 (chrX:154709953:G:C)

	rsid	hgnc_symbol	dataset	tissue	PPH3	PPH4
1	rs5945232	F8	GTEx_eQTL	Brain_Nucleus_accumbens_basal_ganglia	0.352028	0.6477378

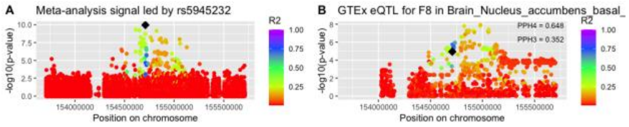

This meta-analysis signal did not strongly colocalize with any QTLs. The proximal gene was *GAB3*. *DKC1* is 52.9 kilobases away and has key roles in telomere length regulation. Therefore, we attribute this signal to *DKC1*.

Supplementary Note 2

Supplementary Note 2 walks through the process of identifying candidate genes for validation experiments nominated by the computational analyses. We provide an R script that walks through this filtering process, supplementary_note2.Rmd, in our code.

Stage 1: Require colocalization with a GTEx eQTL. The molecular mechanisms underlying GWAS signals are varied and we chose to focus on those where there was additional evidence suggesting that changes in expression of the putative causal gene would have an effect on telomere length. While we chose to inform our gene selection first by colocalization analysis with eQTLs, additional approaches could be considered to generate a gene list including colocalization with other QTL datasets (splicing QTLs, protein QTLs, methylation QTLs, etc.) or TWAS. For a gene to meet this stage of the filtering process we required that there was at least moderate colocalization (PPH4 > 0.5) between the meta-analysis signal and a GTEx eQTL in any tissue (Supplementary Data 4). Only meta-analysis signals that were genome-wide significant ($p<5x10^{-8}$) were considered. In the main text we describe a threshold of PPH4 > 0.7 to be strong colocalization. We used a lower threshold for the initial filtering step for selecting genes for validation because colocalization analysis results can be impacted by technical aspects including the power of the eQTL dataset and cell type specific effects (discussed in the Results related to Figure 2C) and since we planned to experimentally validate the nominated genes, we were comfortable lowering the threshold to increase the number of genes we considered. This left us with 82 candidate genes across 35 meta-analysis signals.

Stage 2: Require a positive effect size estimate. We planned to overexpress the candidate genes in the first validation experiment. Therefore we limited the candidates to those that were expected to have an effect on telomere length when their expression was increased. An eQTL may have a positive or negative effect size estimate, that is with increased alternate allele copy number gene expression may increase or decrease (Figure 1). We generated a list of the eQTL effect size estimates for the meta-analysis lead variant at each eQTL that colocalized with a meta-analysis signal (PPH4 > 0.5) in the tissue where colocalization was strongest (highest PPH4). We limited this list to cases where the lead variants had significant effects on gene expression (FDR < 0.05 in GTEx). That is, we took the eQTL - meta-analysis colocalization pairs that had PPH4 > 0.5 in Stage 1 and examined their effect size estimate as an eQTL in GTEx for the gene that colocalized in the tissue where colocalization was strongest. Because we planned to overexpress the genes, we only considered cases where the meta-analysis lead

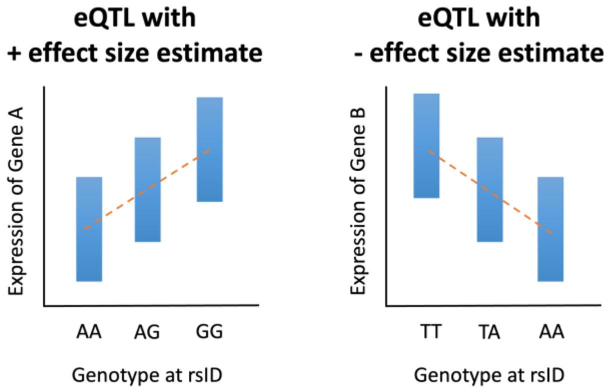

Figure 1: eQTLs are calculated using linear regression between the genotype and gene expression levels (in addition to other covariates). The dotted orange line represents the line of best fit. The slope of this line, the effect size estimate (aka beta), estimates the strength of the effect of the genetic variant on the expression of the tested gene. A larger absolute value of the effect size estimate indicates a stronger effect of the genetic variant on gene expression.

SNP tested allele was also associated with increased gene expression in GTEx, that is that the same allele had a positive effect size estimate. Notably, this includes genes whose overexpression could increase or decrease telomere length (which is predicted by the meta-analysis effect size estimate/beta). This left us with 40 candidate genes across 22 meta-analysis signals.

Stage 3: Require a single known isoform or small genomic coding region. Our overexpression experiment required that the gene be expressed from a plasmid. Therefore, we faced a technical constraint where we would have to be able to fit the coding sequence of the gene in the plasmid. This is often done by inserting the spliced mRNA coding sequence which requires a choice of which transcript to use. As different transcriptional isoforms may have distinct effects on telomere length, we chose to limit our analysis to genes with one reported isoform in the NCBI gene database or with a coding region less than 15 kilobases such that it could be cloned into a plasmid without choosing an isoform. Notably, some of the genes had multiple transcriptional isoforms but the isoforms only differed in the untranslated regions. As this region would not be included in the overexpression cassette, we included genes as passing this filtering step. This was a manual curation that reduced our candidate gene list to 23 genes across 15 meta-analysis signals. Data on all genes that passed stage 2 and were evaluated in stage 3 is provided in Supplementary Note 2 Table 1.

Supplementary Note 2 Table 1:

rsID	Hgnc symbol	GTEx tissue	PPH4	GTEx eQTL pvalue	GTEx eQTL effect size estimate	N isoforms	Hg38 start	Hg38 end	Gene length (bp)
rs12044242	PSMB4	Nerve_Tibial	0.72363949	1.44E-05	0.0787116	1	151399573	151401937	2364
rs3131064	POU5F1	Thyroid	0.89072	0.000447415	0.237683	4	31164337	31170682	6345
rs3131064	CCHCR1	Heart_Left_Ventricle	0.73188725	9.67E-05	0.18303	3	31142439	31158231	15792
rs1150748	VARS2	Pancreas	0.83914016	4.15E-09	0.39565	3	30914238	30926459	12221
rs1150748	MUCL3	Nerve_Tibial	0.61557958	2.67E-05	0.207543	1	30940973	30954221	13248
rs1150748	BAG6	Brain_Hippocampus	0.91341078	2.94E-08	0.378089	4	31639028	31660900	21872
rs1150748	HLA-DQA2	Artery_Aorta	0.69262968	4.78E-08	0.390431	1	32741391	32747198	5807
rs6968500	POT1-AS1	Thyroid	0.93823135	1.23E-09	0.272989	2	124929873	125145234	215361
rs10954213	TNPO3	Brain_Putamen_basal_ganglia	0.56964144	3.19E-06	0.35302	10	128954185	129055111	100926
rs10954213	IRF5	Testis	0.79785614	1.98E-06	0.22422	3	128937032	128950038	13006

rs10954213	TPI1P2	Pituitary	0.65941972	3.39E-05	0.279917	1	129055223	129057239	2016
rs10958468	TGS1	Skin_Sun_Exposed_Lower_leg	0.68763066	1.01E-41	0.581891	3	55773446	55826445	52999
rs10958468	TMEM68	Esophagus_Gastroesophageal_Junction	0.51936363	9.95E-08	0.387417	5	55738758	55773378	34620
rs10111287	VIRMA-DT	Colon_Sigmoid	0.54246388	3.83E-06	0.34623	6	94553713	94570650	16937
rs7923385	RRP12	Brain_Frontal_Cortex_BA9	0.62150165	2.14E-09	0.280988	3	97356357	97401340	44983
rs7923385	RP11-452K12.4	Whole_Blood	0.57681564	1.71E-05	0.129621	NA	NA	NA	NA
rs2475215	STN1	Brain_Putamen_basal_ganglia	0.64150529	1.63E-05	0.396283	1	103877569	103918184	40615
rs74892322	COX6A1	Pituitary	0.62175569	1.74E-06	0.299593	1	120438113	120440730	2617
rs74892322	POP5	Brain_Nucleus_accumbens_basal_ganglia	0.56576441	4.32E-05	0.263901	1	120578764	120581452	2688
rs74892322	GATC	Nerve_Tibial	0.8538557	5.50E-48	0.77321	2	120446444	120463749	17305
rs28755851	ARL6IP4	Stomach	0.59214913	3.39E-11	0.231025	8	122980681	122982909	2228
rs1411041	KBTBD6	Spleen	0.90784437	1.32E-06	0.408958	1	41127569	41132802	5233
rs11623533	CCDC88C	Brain_Frontal_Cortex_BA9	0.650732	2.22E-05	0.259939	1	91271323	91417820	146497
rs12934863	PRMT7	Brain_Anterior_cingulate_cortex_BA24	0.86644404	1.39E-06	0.325074	13	68310982	68360876	49894
rs12934863	SLC9A5	Adipose_Subcutaneous	0.69595203	5.54E-05	0.122678	6	67247549	67272191	24642
rs12934863	DUS2	Cells_Cultured_fibroblasts	0.95922534	6.93E-23	0.184242	2	68023284	68079320	56036
rs12934863	KCTD19	Testis	0.66314879	4.29E-06	0.125263	1	67289432	67326741	37309
rs12934863	B3GNT9	Artery_Tibial	0.62427233	5.12E-07	0.139518	1	67148104	67150998	2894
rs9939870	VPS4A	Muscle_Skeletal	0.53758182	8.95E-05	0.0640718	1	69311350	69326939	15589
rs12149396	CLEC18C	Adrenal_Gland	0.97381726	3.69E-22	0.860509	5	70173322	70187147	13825
rs12149396	RP11-296I10.3	Testis	0.97406387	1.10E-58	1.05444	NA	NA	NA	NA
rs208011	NFE2L1	Thyroid	0.68387017	8.19E-09	0.242825	3	48048359	48061545	13186

rs208011	CBX1	Colon_Transverse	0.6844636	3.14E-09	0.206356	1	48070059	48101478	31419
rs144204502	TK1	Brain_Nucleus_accumbens_basal_ganglia	0.99960311	9.33E-18	1.46518	3	78174079	78187204	13125
rs2124616	RP11-769O8.3	Breast_Mammary_Tissue	0.70413076	2.40E-06	0.335115	NA	NA	NA	NA
rs8105767	ZNF257	Brain_Hypothalamus	0.99926128	1.68E-13	0.720474	3	22052484	22091480	38996
rs79476302	RBL1	Cells_Cultured_fibroblasts	0.59400068	2.28E-07	0.126347	3	36996349	37095997	99648
rs131784	TYMP	Pancreas	0.95744686	2.63E-06	0.155994	2	50525752	50530085	4333
rs131784	LMF2	Brain_Nucleus_accumbens_basal_ganglia	0.64027454	1.39E-05	0.187404	2	50502949	50507781	4832
rs131784	ODF3B	Colon_Transverse	0.9191106	9.25E-06	0.143879	3	50530426	50532498	2072

Stage 4: In the final stage of narrowing down our list of genes for experimental validation, we turned to existing knowledge of telomere biology and gene function. As a collaborative team representing experts in the telomere biology field, we chose five genes to begin our validation experiments based on what was known about their function in the literature. We also considered that genes critical for telomere length maintenance through more direct mechanisms are likely going to be expressed consistently across different cellular contexts since telomere length maintenance must occur in all cells (though telomerase regulation is limited to specific cell types) and so we prioritized genes that are expressed at approximately consistent level across GTEx tissues. In addition, we prioritized genes expressed across diverse cell types to improve the likelihood of detecting the effect of the gene on telomere length in human cell culture. OBFC1 is a known protein component of the CST complex with clear roles in regulating telomere length maintenance, however, we suspected that overexpressing a single component of the complex would not have a strong effect on telomere length. POP5 is part of the Ribonuclease P/MRP complex and we were very interested in this candidate gene because the Ribonuclease P/MRP complex in *S. cerevisiae* was previously shown to be involved in the biosynthesis of the RNA component of telomerase [27]. CBX1 is part of the heterochromatin regulation machinery and as telomeres are maintained in a heterochromatic state, this candidate gene was interesting to us. Finally, both PSMB4 and KBTBD6 are, like OBFC1, part of protein complexes. We prioritized these because of their stronger colocalization results and because we noted there were several genes associated with telomere length that are involved in ubiquitin-dependent protein degradation and thought it would be interesting to see whether we could detect a role for these proteins in telomere length regulation through this experiment. We intended to also overexpress the genomic regions for *TYMP* and *ODF3B*, which have a genomic region less than 15 kilobases, but were unable to identify a manufacturer with the bacterial artificial chromosomes containing these genes.

Below is a synopsis of the computational data supporting the five genes we overexpressed.

PSMB4

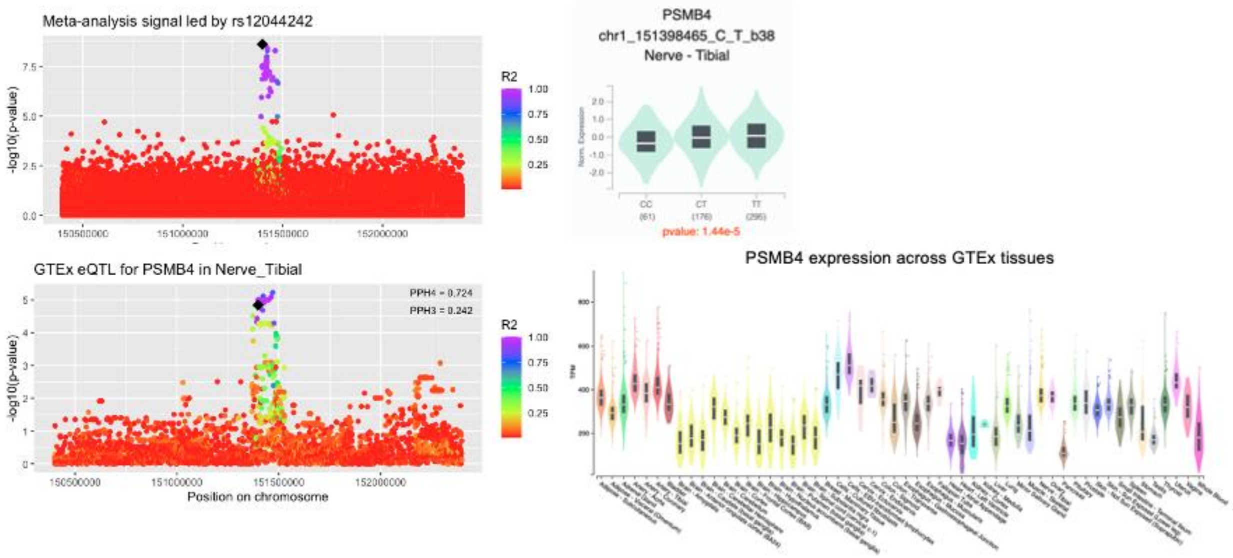

OBFC1 (STN1)

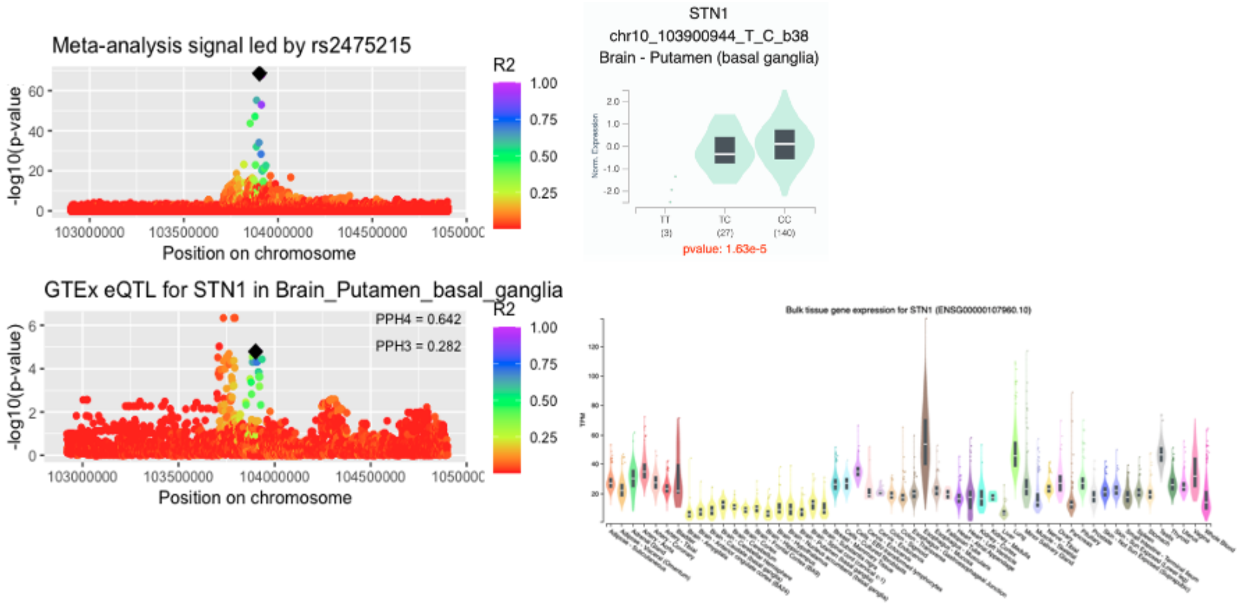

KBTBD6

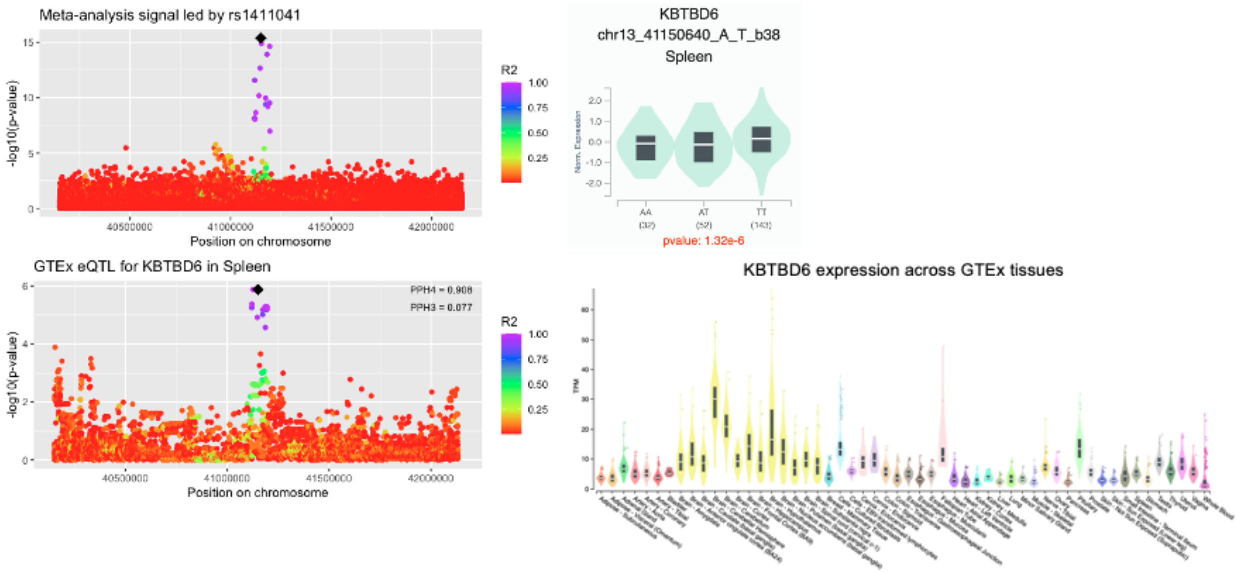
Meta-analysis signal led by rs1411041
-log10(p-value)
Position on chromosome
R2
1.00
0.75
0.50
0.25
KBTBD6
chr13_41150640_A_T_b38
Spleen
pvalue: 1.32e-6
GTEx eQTL for KBTBD6 in Spleen
PPH4 = 0.908
PPH3 = 0.077
KBTBD6 expression across GTEx tissues
TPM

CBX1

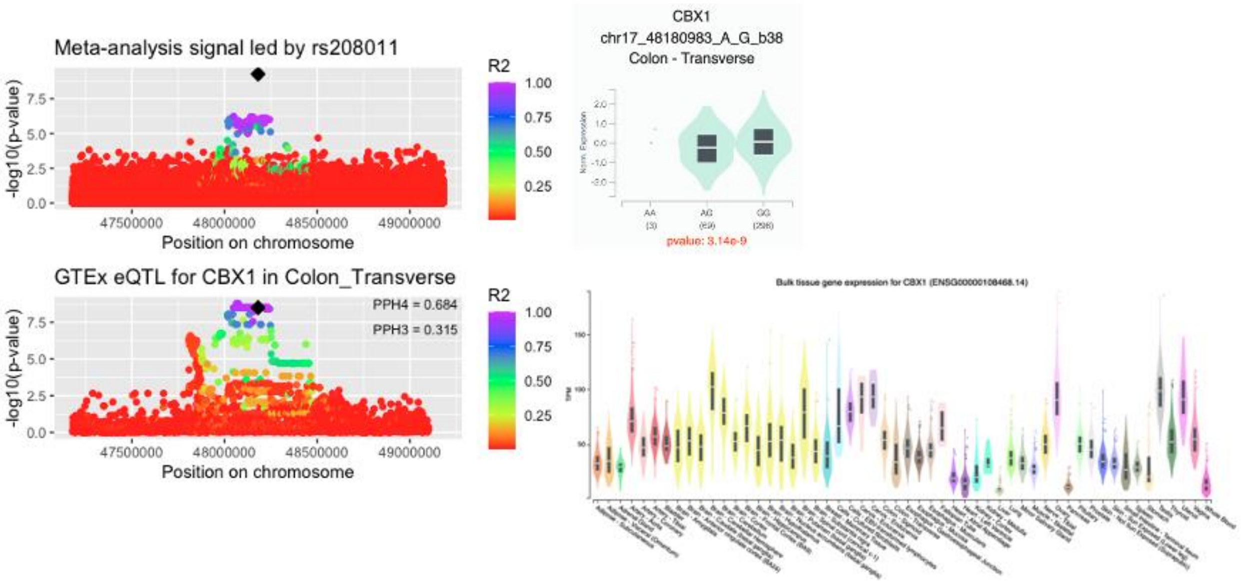
Meta-analysis signal led by rs208011
-log10(p-value)
Position on chromosome
R2
1.00
0.75
0.50
0.25
CBX1
chr17_48180983_A_G_b38
Colon - Transverse
pvalue: 3.14e-9
GTEx eQTL for CBX1 in Colon_Transverse
PPH4 = 0.684
PPH3 = 0.315

POP5

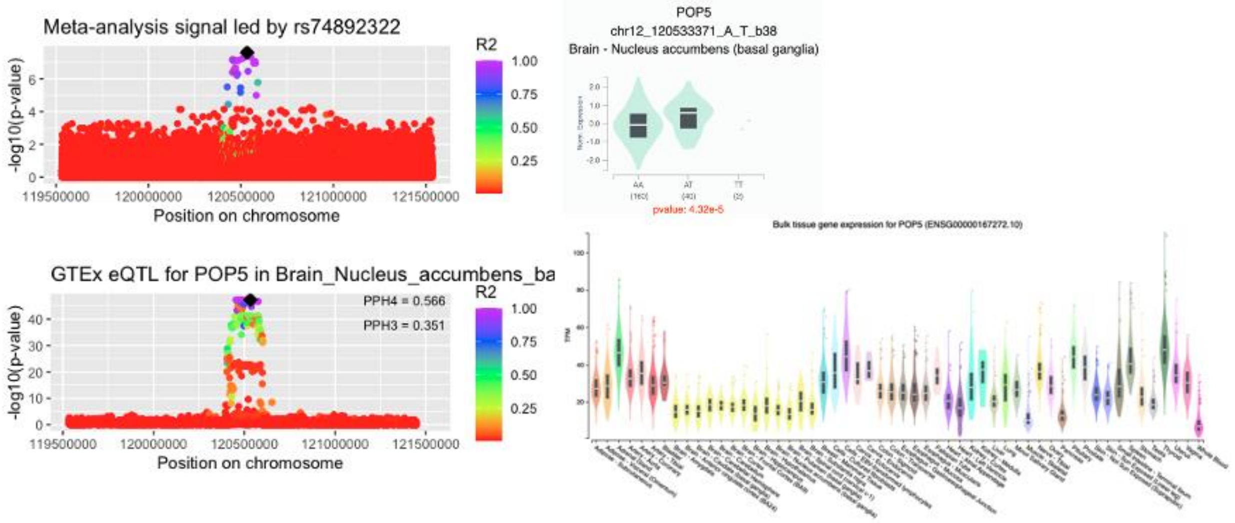

Replication of POP5 locus colocalization using Codd et al. 2021 summary statistics
The summary statistics from Codd et al. 2021 were used in colocalization analysis with GTEx eQTLs.

**Hgnc symbol**	**dataset**	**tissue**	**Codd et al. 2021 UKBB telomere length GWAS colocalized with GTEx eQTLs**		**Keener et al. meta-analysis colocalized with GTEx eQTLs**	
			**PPH3**	**PPH4**	**PPH3**	**PPH4**
POP5	GTEx eQTL	Brain_Nucleus_accumbens_basal_ganglia	0.391	0.532	0.351	0.565
GATC	GTEx eQTL	Skin_Not_Sun_Exposed_Suprapublic	0.199	0.801	0.145	0.853
SRSF9	GTEx eQTL	Testis	0.269	0.664	0.367	0.534
COX6A1	GTEx eQTL	Pituitary	0.475	0.518	0.370	0.621

The signal led by rs74892322 in Keener et al. strongly colocalized with a signal near rs74892322 in the Codd et al. 2021 analysis.

Colocalization analysis between the two sets of GWAS summary statistics.

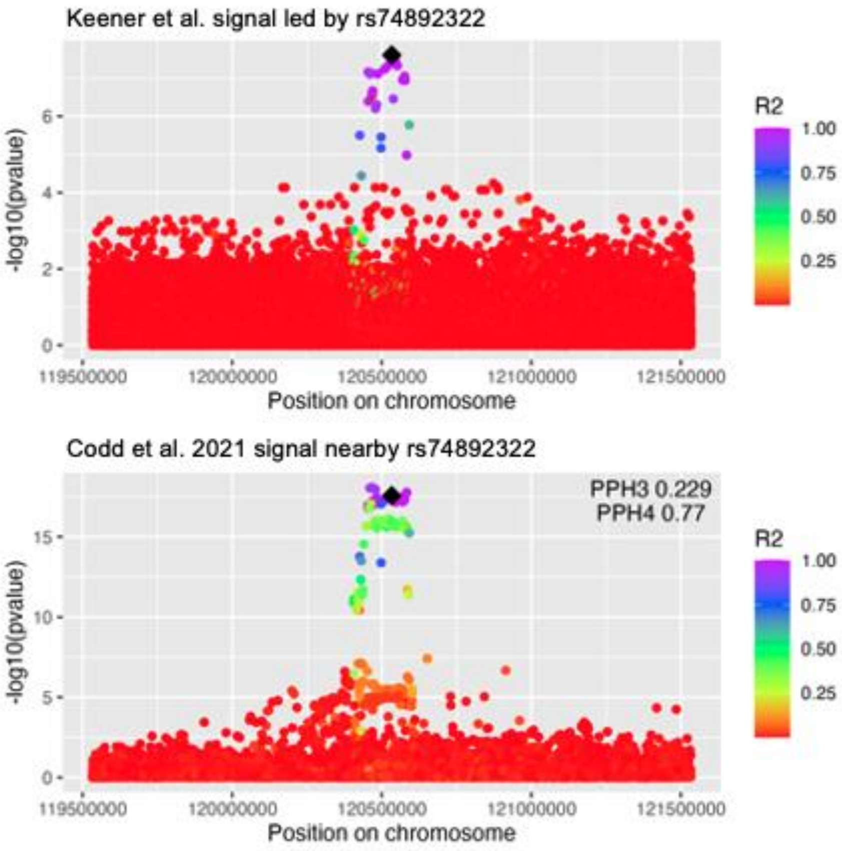

Supplementary Acknowledgements

Generation of TOPMed whole genome sequencing data by study

Whole genome sequencing (WGS) for the Trans-Omics in Precision Medicine (TOPMed) program was supported by the National Heart, Lung and Blood Institute (NHLBI). WGS for NHLBI TOPMed: AFLMU (phs001543) was performed at Broad Genomics (3UM1HG008895-01S2; HHSN268201500014C); WGS for NHLBI TOPMed: Amish (phs000956) was performed at Broad Genomics (3R01HL121007-01S1); WGS for NHLBI TOPMed: ARIC (phs001211) was performed at Baylor (3U54HG003273-12S2 / HHSN268201500015C,3R01HL092577-06S1), Broad Genomics (3U54HG003273-12S2 / HHSN268201500015C,3R01HL092577-06S1); WGS for NHLBI TOPMed: BioMe (phs001644) was performed at MGI (HHSN268201600037I,HHSN268201600033I,3UM1HG008853-01S2), Baylor (HHSN268201600037I,HHSN268201600033I,3UM1HG008853-01S2); WGS for NHLBI TOPMed: CAMP (phs001726) was performed at NWGC (HHSN268201600032I); WGS for NHLBI TOPMed: CARDIA (phs001612) was performed at Baylor (HHSN268201600033I); WGS for NHLBI TOPMed: CARE_BADGER (phs001728) was performed at NWGC (HHSN268201600032I); WGS for NHLBI TOPMed: CARE_CLIC (phs001729) was performed at NWGC (HHSN268201600032I); WGS for NHLBI TOPMed: CARE_PACT (phs001730) was performed at NWGC (HHSN268201600032I); WGS for NHLBI TOPMed: CARE_TREXA (phs001732) was performed at NWGC (HHSN268201600032I); WGS for NHLBI TOPMed: CFS (phs000954) was performed at NWGC (HHSN268201600032I,3R01HL098433-05S1); WGS for NHLBI TOPMed: ChildrensHS_GAP (phs001602) was performed at NWGC (HHSN268201600032I); WGS for NHLBI TOPMed: ChildrensHS_IGERA (phs001603) was performed at NWGC (HHSN268201600032I); WGS for NHLBI TOPMed: ChildrensHS_MetaAir (phs001604) was performed at NWGC (HHSN268201600032I); WGS for NHLBI TOPMed: CHIRAH (phs001605) was performed at NWGC (HHSN268201600032I); WGS for NHLBI TOPMed: CHS (phs001368) was performed at Baylor (HHSN268201600033I,3U54HG003273-12S2 / HHSN268201500015C); WGS for NHLBI TOPMed: COPDGene (phs000951) was performed at NWGC (3R01HL089856-08S1,HHSN268201500014C), Broad Genomics (3R01HL089856-08S1,HHSN268201500014C); WGS for NHLBI TOPMed: CRA (phs000988) was performed at NWGC (3R37HL066289-13S1,HHSN268201600032I); WGS for NHLBI TOPMed: DHS (phs001412) was performed at Broad Genomics (HHSN268201500014C); WGS for NHLBI TOPMed: ECLIPSE (phs001472) was performed at MGI (HHSN268201600037I); WGS for NHLBI TOPMed: EOCOPD (phs000946) was performed at NWGC (3R01HL089856-08S1); WGS for NHLBI TOPMed: FHS (phs000974) was performed at Broad Genomics (3U54HG003067- 12S2,3R01HL092577-06S1); WGS for NHLBI TOPMed: GALAI (phs001542) was performed at NWGC (HHSN268201600032I); WGS for NHLBI TOPMed: GALAII (phs000920) was performed at NYGC (3R01HL117004-02S3,HHSN268201600032I), NWGC (3R01HL117004-02S3,HHSN268201600032I), NYGC (UM1 HG008901); WGS for NHLBI TOPMed: GeneSTAR (phs001218) was performed at Psomagen (3R01HL112064-04S1,R01HL112064,HHSN268201500014C), Illumina (3R01HL112064-04S1,R01HL112064,HHSN268201500014C), Broad Genomics (3R01HL112064-04S1,R01HL112064,HHSN268201500014C); WGS for NHLBI TOPMed: GENOA (phs001345)

was performed at NWGC (3R01HL055673-18S1,HHSN268201500014C), Broad Genomics (3R01HL055673-18S1,HHSN268201500014C); WGS for NHLBI TOPMed: GenSalt (phs001217) was performed at Baylor (HHSN268201500015C); WGS for NHLBI TOPMed: GOLDN (phs001359) was performed at NWGC (3R01HL104135-04S1); WGS for NHLBI TOPMed: HCHS/SOL (phs001395) was performed at Baylor College of Medicine Human Genome Sequencing Center (HHSN268201600033I); WGS for NHLBI TOPMed: HVH (phs000993) was performed at Broad Genomics (3R01HL092577-06S1,3U54HG003273-12S2 / HHSN268201500015C), Baylor (3R01HL092577-06S1,3U54HG003273-12S2 / HHSN268201500015C); WGS for NHLBI TOPMed: HyperGEN (phs001293) was performed at NWGC (3R01HL055673-18S1); WGS for NHLBI TOPMed: IPF (phs001607) was performed at MGI (HHSN268201600037I); WGS for NHLBI TOPMed: JHS (phs000964) was performed at NWGC (HHSN268201100037C); WGS for NHLBI TOPMed: LTRC (phs001662) was performed at Broad Genomics (HHSN268201600034I); WGS for NHLBI TOPMed: Mayo_VTE (phs001402) was performed at Baylor (3U54HG003273-12S2 / HHSN268201500015C); WGS for NHLBI TOPMed: MESA (phs001416) was performed at Broad Genomics (3U54HG003067-13S1,HHSN268201500014C); WGS for NHLBI TOPMed: MLOF (phs001515) was performed at Baylor (HHSN268201600033I,HHSN268201500016C), NYGC (HHSN268201600033I,HHSN268201500016C); WGS for NHLBI TOPMed: OMG_SCD (phs001608) was performed at Baylor (HHSN268201500015C); WGS for NHLBI TOPMed: PCGC_CHD (phs001735) was performed at Broad Genomics (HHSN268201600034I); WGS for NHLBI TOPMed: PharmHU (phs001466) was performed at Baylor (HHSN268201500015C); WGS for NHLBI TOPMed: PIMA (phs001727) was performed at NWGC (HHSN268201600032I); WGS for NHLBI TOPMed: PUSH_SCD (phs001682) was performed at Baylor (HHSN268201500015C); WGS for NHLBI TOPMed: REDS-III_Brazil (phs001468) was performed at Baylor (HHSN268201500015C); WGS for NHLBI TOPMed: SAFS (phs001215) was performed at Illumina (R01HL113322,3R01HL113323-03S1); WGS for NHLBI TOPMed: SAGE (phs000921) was performed at NYGC (3R01HL117004- 02S3,HHSN268201600032I), NWGC (3R01HL117004-02S3,HHSN268201600032I); WGS for NHLBI TOPMed: SAPPHIRE_asthma (phs001467) was performed at NWGC (HHSN268201600032I); WGS for NHLBI TOPMed: SARP (phs001446) was performed at NYGC (HHSN268201500016C); NHLBI TOPMed: SAS (phs000972) was performed at NWGC (HHSN268201100037C,HHSN268201500016C), NYGC (HHSN268201100037C,HHSN268201500016C); WGS for NHLBI TOPMed: THRV (phs001387) was performed at Baylor (3R01HL111249-04S1 / HHSN26820150015C); WGS for NHLBI TOPMed: VAFAR (phs000997) was performed at Broad Genomics (3U54HG003067-12S2 / 3U54HG003067- 13S1; 3UM1HG008895-01S2; 3UM1HG008895-01S2,3R01HL092577-06S1); WGS for NHLBI TOPMed: VU_AF (phs001032) was performed at Broad Genomics (3R01HL092577-06S1); WGS for NHLBI TOPMed: walk_PHaSST (phs001514) was performed at Baylor (HHSN268201500015C); WGS for NHLBI TOPMed: WGHS (phs001040) was performed at Broad Genomics (3R01HL092577-06S1); WGS for NHLBI TOPMed: WHI (phs001237) was performed at Broad Genomics (HHSN268201500014C). Core support including centralized genomic read mapping and genotype calling, along with variant quality metrics and filtering were provided by the TOPMed Informatics Research Center (3R01HL-117626-02S1; contract HHSN268201800002I). Core support including phenotype

harmonization, data management, sample-identity QC, and general program coordination were provided by the TOPMed Data Coordinating Center (R01HL-120393; U01HL-120393; contract HHSN268201800001I). We gratefully acknowledge the studies and participants who provided biological samples and data for TOPMed. NYGC = New York Genome Center; Broad Genomics = Broad Institute Genomics Platform; NWGC = University of Washington Northwest Genomics Center; Illumina = Illumina Genomic Services; Psomagen = Psomagen Corp.; Baylor = Baylor Human Genome Sequencing Center; MGI = McDonnell Genome Institute

Study-specific acknowledgements

NHLBI TOPMed: Atrial Fibrillation Biobank LMU (AFLMU) in the context of the ArrhythmiaBiobank-LMU
AFLMU is a repository of AF patients recruited in the context of the German Competence Network for Atrial Fibrillation (AFNET) and at the Department of Medicine I of the University Hospital Munich. In this context, DNA samples were preferentially sampled if the patient developed AF before the age of 60 years. Cases were selected if the diagnosis of atrial fibrillation was made on an electrocardiogram analyzed by a trained physician. Patients with signs of moderate to severe heart failure, moderate to severe valve disease or with hyperthyroidism were excluded from the study. All participants provided written informed consent. AFLMU was approved by the Ethics Committee at the Ludwig-Maximilian's University.

NHLBI TOPMed: Genetics of Cardiometabolic Health in the Amish (Amish)
The Amish studies upon which these data are based were supported by NIH grants R01 AG18728, U01 HL072515, R01 HL088119, R01 HL121007, and P30 DK072488. See publication: PMID: 18440328

NHLBI TOPMed: Atherosclerosis Risk in Communities (ARIC)
The Atherosclerosis Risk in Communities study has been funded in whole or in part with Federal funds from the National Heart, Lung, and Blood Institute, National Institutes of Health, Department of Health and Human Services (contract numbers HHSN268201700001I, HHSN268201700002I, HHSN268201700003I, HHSN268201700004I and HHSN268201700005I). The authors thank the staff and participants of the ARIC study for their important contributions.

NHLBI TOPMed: The Genetics and Epidemiology of Asthma in Barbados (BAGS)
We gratefully acknowledge the contributions of Pissamai and Trevor Maul, Paul Levett, Anselm Hennis, P. Michele Lashley, Raana Naidu, Malcolm Howitt and Timothy Roach, and the numerous health care providers, and community clinics and co-investigators who assisted in the phenotyping and collection of DNA samples, and the families and patients for generously donating DNA samples to the Barbados Asthma Genetics Study (BAGS). The Genetics and Epidemiology of Asthma in Barbados is supported by National Institutes of Health (NIH) National Heart, Lung, Blood Institute TOPMed (R01 HL104608-S1) and: R01 AI20059, K23 HL076322, R01HL087699, and RC2 HL101651. For the specific cohort descriptions and descriptions regarding the collection of phenotype data can be found at: https://www.nhlbiwgs.org/group/bags-asthma. The authors wish to give special recognition to

the individual study participants who provided biological samples and or data, without their support in research none of this would be possible.

NHLBI TOPMed: BioMe Biobank at Mount Sinai (BioMe)
The Mount Sinai BioMe Biobank has been supported by The Andrea and Charles Bronfman Philanthropies and in part by Federal funds from the NHLBI and NHGRI (U01HG00638001; U01HG007417; X01HL134588). We thank all participants in the Mount Sinai Biobank. We also thank all our recruiters who have assisted and continue to assist in data collection and management and are grateful for the computational resources and staff expertise provided by Scientific Computing at the Icahn School of Medicine at Mount Sinai.

NHLBI TOPMed: CAMP
We thank the clinical centers and the Data Coordinating Center of the Childhood Asthma Management Program (CAMP) as well as all of the study participants at the 8 clinical sites. The CAMP study was supported by NHLBI P01 HL132825.

NHLBI TOPMed: Coronary Artery Risk Development in Young Adults Study (CARDIA)
The Coronary Artery Risk Development in Young Adults Study (CARDIA) is conducted and supported by the National Heart, Lung, and Blood Institute (NHLBI) in collaboration with the University of Alabama at Birmingham (HHSN268201800005I & HHSN268201800007I), Northwestern University (HHSN268201800003I), University of Minnesota (HHSN268201800006I), and Kaiser Foundation Research Institute (HHSN268201800004I). CARDIA was also partially supported by the Intramural Research Program of the National Institute on Aging (NIA) and an intra-agency agreement between NIA and NHLBI (AG0005).

NHLBI TOPMed: CARE_BADGER
This research was supported by grants from the National Heart, Lung, and Blood Institute (NHLBI), ((5U10HL064287, 5U10HL064288, 5U10HL064295, 5U10HL064307, 5U10HL064305, 5U10HL064313, and HL080083)

NHLBI TOPMed: CARE_CLIC
This research was supported by grants from the National Heart, Lung, and Blood Institute (NHLBI), ((5U10HL064287, 5U10HL064288, 5U10HL064295, 5U10HL064307, 5U10HL064305, 5U10HL064313, and HL080083)

NHLBI TOPMed: CARE_PACT
This research was supported by grants from the National Heart, Lung, and Blood Institute (NHLBI), ((5U10HL064287, 5U10HL064288, 5U10HL064295, 5U10HL064307, 5U10HL064305, 5U10HL064313, and HL080083)

NHLBI TOPMed: CARE_TREXA
This research was supported by grants from the National Heart, Lung, and Blood Institute (NHLBI), ((5U10HL064287, 5U10HL064288, 5U10HL064295, 5U10HL064307, 5U10HL064305, 5U10HL064313, and HL080083)

NHLBI TOPMed: The Cleveland Family Study (CFS)
The Cleveland Family Study has been supported in part by National Institutes of Health grants [R01- HL046380, KL2-RR024990, R35-HL135818, and R01-HL113338].

NHLBI TOPMed: Children's Health Study: Integrative Genetic Approaches to Gene-Air Pollution Interactions in Asthma (ChildrensHS_GAP)
The Integrative Genetic Approaches to Gene-Air Pollution Interactions in Asthma (GAP) study was supported by the National Institute of Environmental Health Sciences (NIEHS) grant # R01ES021801. The Children's Health Study (CHS) was supported by the Southern California Environmental Health Sciences Center (grant P30ES007048); National Institute of Environmental Health Sciences (grants 5P01ES011627, ES021801, ES023262, P01ES009581, P01ES011627, P01ES022845, R01 ES016535, R03ES014046, P50 CA180905, R01HL061768, R01HL076647, R01HL087680 and RC2HL101651), the Environmental Protection Agency (grants RD83544101, R826708, RD831861, and R831845), and the Hastings Foundation.

NHLBI TOPMed: Children's Health Study: Integrative Genomics and Environmental Research of Asthma (ChildrensHS_IGERA)
The Integrative Genomics and Environmental Research of Asthma (IGERA) Study was supported by the National Heart, Lung and Blood Institute (grant # RC2HL101543 -The Asthma BioRepository for Integrative Genomics Research, PI Gilliland/Raby). The Children's Health Study (CHS) was supported by the Southern California Environmental Health Sciences Center (grant P30ES007048); National Institute of Environmental Health Sciences (grants 5P01ES011627, ES021801, ES023262, P01ES009581, P01ES011627, P01ES022845, R01 ES016535, R03ES014046, P50 CA180905, R01HL061768, R01HL076647, R01HL087680 and RC2HL101651), the Environmental Protection Agency (grants RD83544101, R826708, RD831861, and R831845), and the Hastings Foundation.

NHLBI TOPMed: Children's Health Study: Effects of Air Pollution on the Development of Obesity in Children (ChildrensHS_MetaAir)
The Effects of Air Pollution on the Development of Obesity in Children (Meta-AIR) study was supported by the Southern California Children’s Environmental Health Center funded by the National Institute of Environmental Health Sciences (NIEHS) (P01ES022845) and the Environmental Protection Agency (EPA) (RD-83544101–0). The Children's Health Study (CHS) was supported by the Southern California Environmental Health Sciences Center (grant P30ES007048); National Institute of Environmental Health Sciences (grants 5P01ES011627, ES021801, ES023262, P01ES009581, P01ES011627, P01ES022845, R01 ES016535, R03ES014046, P50 CA180905, R01HL061768, R01HL076647, R01HL087680 and RC2HL101651), the Environmental Protection Agency (grants RD83544101, R826708, RD831861, and R831845), and the Hastings Foundation.

NHLBI TOPMed: Genetics Sub-Study of Chicago Initiative to Raise Asthma Health Equity (CHIRAH)

Support for the Genetics Sub-Study of Chicago Initiative to Raise Asthma Health Equity was provided by NHLBI grant number UO1 HL072496.

NHLBI TOPMed: Cardiovascular Health Study (CHS)
This research was supported by contracts HHSN268201200036C, HHSN268200800007C, HHSN268201800001C, N01HC55222, N01HC85079, N01HC85080, N01HC85081, N01HC85082, N01HC85083, N01HC85086, and 75N92021D00006, and grants U01HL080295 and U01HL130114 from the National Heart, Lung, and Blood Institute (NHLBI), with additional contribution from the National Institute of Neurological Disorders and Stroke (NINDS). Additional support was provided by R01AG023629 from the National Institute on Aging (NIA). A full list of principal CHS investigators and institutions can be found at CHS-NHLBI.org. The content is solely the responsibility of the authors and does not necessarily represent the official views of the National Institutes of Health.

NHLBI TOPMed: Genetic Epidemiology of COPD (COPDGene) in the TOPMed Program
The COPDGene project described was supported by Award Number U01 HL089897 and Award Number U01 HL089856 from the National Heart, Lung, and Blood Institute. The content is solely the responsibility of the authors and does not necessarily represent the official views of the National Heart, Lung, and Blood Institute or the National Institutes of Health. The COPDGene project is also supported by the COPD Foundation through contributions made to an Industry Advisory Board comprised of AstraZeneca, Boehringer Ingelheim, GlaxoSmithKline, Novartis, Pfizer, Siemens and Sunovion. A full listing of COPDGene investigators can be found at: http://www.copdgene.org/directory

NHLBI TOPMed: The Genetic Epidemiology of Asthma in Costa Rica (CRA)
This study was supported by NHLBI grants R37 HL066289 and P01 HL132825. We wish to acknowledge the investigators at the Channing Division of Network Medicine at Brigham and Women's Hospital, the investigators at the Hospital Nacional de Niños in San José, Costa Rica and the study subjects and their extended family members who contributed samples and genotypes to the study, and the NIH/NHLBI for its support in making this project possible.

NHLBI TOPMed: Diabetes Heart Study (DHS)
This work was supported by R01 HL92301, R01 HL67348, R01 NS058700, R01 AR48797, R01 DK071891, R01 AG058921, the General Clinical Research Center of the Wake Forest University School of Medicine (M01 RR07122, F32 HL085989), the American Diabetes Association, and a pilot grant from the Claude Pepper Older Americans Independence Center of Wake Forest University Health Sciences (P60 AG10484).

NHLBI TOPMed: ECLIPSE
The ECLIPSE study (NCT00292552) was sponsored by GlaxoSmithKline. The ECLIPSE investigators included: ECLIPSE Investigators — Bulgaria: Y. Ivanov, Pleven; K. Kostov, Sofia. Canada: J. Bourbeau, Montreal; M. Fitzgerald, Vancouver, BC; P. Hernandez, Halifax, NS; K. Killian, Hamilton, ON; R. Levy, Vancouver, BC; F. Maltais, Montreal; D. O'Donnell, Kingston, ON. Czech Republic: J. Krepelka, Prague. Denmark: J. Vestbo, Hvidovre. The Netherlands: E.

Wouters, Horn-Maastricht. New Zealand: D. Quinn, Wellington. Norway: P. Bakke, Bergen. Slovenia: M. Kosnik, Golnik. Spain: A. Agusti, J. Sauleda, P. de Mallorca. Ukraine: Y. Feschenko, V. Gavrisyuk, L. Yashina, Kiev; N. Monogarova, Donetsk. United Kingdom: P. Calverley, Liverpool; D. Lomas, Cambridge; W. MacNee, Edinburgh; D. Singh, Manchester; J. Wedzicha, London. United States: A. Anzueto, San Antonio, TX; S. Braman, Providence, RI; R. Casaburi, Torrance CA; B. Celli, Boston; G. Giessel, Richmond, VA; M. Gotfried, Phoenix, AZ; G. Greenwald, Rancho Mirage, CA; N. Hanania, Houston; D. Mahler, Lebanon, NH; B. Make, Denver; S. Rennard, Omaha, NE; C. Rochester, New Haven, CT; P. Scanlon, Rochester, MN; D. Schuller, Omaha, NE; F. Sciurba, Pittsburgh; A. Sharafkhaneh, Houston; T. Siler, St. Charles, MO; E. Silverman, Boston; A. Wanner, Miami; R. Wise, Baltimore; R. ZuWallack, Hartford, CT. ECLIPSE Steering Committee: H. Coxson (Canada), C. Crim (GlaxoSmithKline, USA), L. Edwards (GlaxoSmithKline, USA), D. Lomas (UK), W. MacNee (UK), E. Silverman (USA), R. Tal-Singer (Co-chair, GlaxoSmithKline, USA), J. Vestbo (Co-chair, Denmark), J. Yates (GlaxoSmithKline, USA). ECLIPSE Scientific Committee: A. Agusti (Spain), P. Calverley (UK), B. Celli (USA), C. Crim (GlaxoSmithKline, USA), B. Miller (GlaxoSmithKline, USA), W. MacNee (Chair, UK), S. Rennard (USA), R. Tal-Singer (GlaxoSmithKline, USA), E. Wouters (The Netherlands), J. Yates (GlaxoSmithKline, USA).

NHLBI TOPMed: Boston Early-Onset COPD Study in the TOPMed Program (EOCOPD)
The Boston Early-Onset COPD Study was supported by R01 HL113264 and U01 HL089856 from the National Heart, Lung, and Blood Institute.

NHLBI TOPMed: Whole Genome Sequencing and Related Phenotypes in the Framingham Heart Study (FHS)
The Framingham Heart Study (FHS) acknowledges the support of contracts NO1-HC-25195, HHSN268201500001I, and 75N92019D00031 from the National Heart, Lung and Blood Institute and grant supplement R01 HL092577-06S1 for this research. We also acknowledge the dedication of the FHS study participants without whom this research would not be possible.

NHLBI TOPMed: Genes-environments and Admixture in Latino Asthmatics (GALA I) Study
The Genes-environments and Admixture in Latino Americans (GALA I) Study was supported by the National Heart, Lung, and Blood Institute of the National Institute of Health (NIH) grants R01HL117004 and X01HL134589; study enrollment supported by Sandler Center for Basic Research in Asthma and the Sandler Family Foundation, the American Asthma Foundation, the American Lung Association, the NIH grants K23HL04464 and HL07185, the Resource Centers for Minority Aging Research from the National Institute on Aging, RCMAR P30-AG15272, the National Institute of Nursing Research and the National Center on Minority Health and Health Disparities.

NHLBI TOPMed: Genes-environments and Admixture in Latino Asthmatics (GALA II) Study
The Genes-environments and Admixture in Latino Americans (GALA II) Study was supported by the National Heart, Lung, and Blood Institute of the National Institute of Health (NIH) grants

R01HL117004 and X01HL134589; study enrollment supported by the Sandler Family Foundation, the American Asthma Foundation, the RWJF Amos Medical Faculty Development Program, Harry Wm. and Diana V. Hind Distinguished Professor in Pharmaceutical Sciences II and the National Institute of Environmental Health Sciences grant R01ES015794 . WGS of part of GALA II was performed by New York Genome Center under The Centers for Common Disease Genomics of the Genome Sequencing Program (GSP) Grant (UM1 HG008901). The GSP Coordinating Center (U24 HG008956) contributed to cross-program scientific initiatives and provided logistical and general study coordination. GSP is funded by the National Human Genome Research Institute, the National Heart, Lung, and Blood Institute, and the National Eye Institute. The GALA II study collaborators include Shannon Thyne, UCSF; Harold J. Farber, Texas Children's Hospital; Denise Serebrisky, Jacobi Medical Center; Rajesh Kumar, Lurie Children's Hospital of Chicago; Emerita Brigino-Buenaventura, Kaiser Permanente; Michael A. LeNoir, Bay Area Pediatrics; Kelley Meade, UCSF Benioff Children’s Hospital, Oakland; William Rodriguez-Cintron, VA Hospital, Puerto Rico; Pedro C. Avila, Northwestern University; Jose R. Rodriguez-Santana, Centro de Neumologia Pediatrica; Luisa N. Borrell, City University of New York; Adam Davis, UCSF Benioff Children's Hospital, Oakland; Saunak Sen, University of Tennessee and Fred Lurmann, Sonoma Technologies, Inc. The authors acknowledge the families and patients for their participation and thank the numerous health care providers and community clinics for their support and participation in GALA II. In particular, the authors thank study coordinator Sandra Salazar; the recruiters who obtained the data: Duanny Alva, MD, Gaby Ayala-Rodriguez, Lisa Caine, Elizabeth Castellanos, Jaime Colon, Denise DeJesus, Blanca Lopez, Brenda Lopez, MD, Louis Martos, Vivian Medina, Juana Olivo, Mario Peralta, Esther Pomares, MD, Jihan Quraishi, Johanna Rodriguez, Shahdad Saeedi, Dean Soto, Ana Taveras; and the lab researcher Celeste Eng who processed the biospecimens.

NHLBI TOPMed: GeneSTAR (Genetic Study of Atherosclerosis Risk)
The Johns Hopkins Genetic Study of Atherosclerosis Risk (GeneSTAR) was supported by grants from the National Institutes of Health through the National Heart, Lung, and Blood Institute (U01HL72518, HL087698, HL112064) and by a grant from the National Center for Research Resources (M01- RR000052) to the Johns Hopkins General Clinical Research Center. We would like to thank the participants and families of GeneSTAR and our dedicated staff for all their sacrifices.

NHLBI TOPMed: Genetic Epidemiology Network of Arteriopathy (GENOA)
Support for GENOA was provided by the National Heart, Lung and Blood Institute (HL054457, HL054464, HL054481, HL119443, and HL087660) of the National Institutes of Health.

NHLBI TOPMed: Genetic Epidemiology Network of Salt Sensitivity (GenSalt)
The Genetic Epidemiology Network of Salt-Sensitivity (GenSalt) was supported by research grants (U01HL072507, R01HL087263, and R01HL090682) from the National Heart, Lung and Blood Institute, National Institutes of Health, Bethesda, MD.

NHLBI TOPMed: Genetics of Lipid Lowering Drugs and Diet Network (GOLDN)

GOLDN biospecimens, baseline phenotype data, and intervention phenotype data were collected with funding from National Heart, Lung and Blood Institute (NHLBI) grant U01 HL072524. Whole-genome sequencing in GOLDN was funded by NHLBI grant R01 HL104135 and supplement R01 HL104135- 04S1.

NHLBI TOPMed: Hispanic Community Health Study/Study of Latinos (HCHS_SOL)
The Hispanic Community Health Study/Study of Latinos is a collaborative study supported by contracts from the National Heart, Lung, and Blood Institute (NHLBI) to the University of North Carolina (HHSN268201300001I / N01-HC-65233), University of Miami (HHSN268201300004I / N01-HC65234), Albert Einstein College of Medicine (HHSN268201300002I / N01-HC-65235), University of Illinois at Chicago – HHSN268201300003I / N01-HC-65236 Northwestern Univ), and San Diego State University (HHSN268201300005I / N01-HC-65237). The following Institutes/Centers/Offices have contributed to the HCHS/SOL through a transfer of funds to the NHLBI: National Institute on Minority Health and Health Disparities, National Institute on Deafness and Other Communication Disorders, National Institute of Dental and Craniofacial Research, National Institute of Diabetes and Digestive and Kidney Diseases, National Institute of Neurological Disorders and Stroke, NIH Institution-Office of Dietary Supplements.

NHLBI TOPMed: Heart and Vascular Health Study (HVH)
The Heart and Vascular Health Study was supported by grants HL068986, HL085251, HL095080, and HL073410 from the National Heart, Lung, and Blood Institute.

NHLBI TOPMed: Hypertension Genetic Epidemiology Network (HyperGEN)
The HyperGEN Study is part of the National Heart, Lung, and Blood Institute (NHLBI) Family Blood Pressure Program; collection of the data represented here was supported by grants U01 HL054472 (MN Lab), U01 HL054473 (DCC), U01 HL054495 (AL FC), and U01 HL054509 (NC FC). The HyperGEN: Genetics of Left Ventricular Hypertrophy Study was supported by NHLBI grant R01 HL055673 with whole-genome sequencing made possible by supplement -18S1.

NHLBI TOPMed: IPF
This research was supported by the National Heart, Lung and Blood Institute (R01-HL097163, P01- HL092870, and UH3-HL123442) and the Department of Defense (W81XWH-17-1-0597).

NHLBI TOPMed: The Jackson Heart Study (JHS)
The Jackson Heart Study (JHS) is supported and conducted in collaboration with Jackson State University (HHSN268201800013I), Tougaloo College (HHSN268201800014I), the Mississippi State Department of Health (HHSN268201800015I) and the University of Mississippi Medical Center (HHSN268201800010I, HHSN268201800011I and HHSN268201800012I) contracts from the National Heart, Lung, and Blood Institute (NHLBI) and the National Institute on Minority Health and Health Disparities (NIMHD). The authors also wish to thank the staffs and participants of the JHS.

NHLBI TOPMed: LTRC

This study utilized biological specimens and data provided by the Lung Tissue Research Consortium (LTRC) supported by the National Heart, Lung, and Blood Institute (NHLBI). The LTRC was sponsored by a contract from the

NHLBI: HHSN2682016000021 NHLBI TOPMed: Mayo Clinic Venous Thromboembolism Study (Mayo_VTE)

Funded, in part, by grants from the National Institutes of Health, National Heart, Lung and Blood Institute (HL66216 and HL83141). the National Human Genome Research Institute (HG04735, HG06379), and research support provided by Mayo Foundation.

NHLBI TOPMed: Multi-Ethnic Study of Atherosclerosis (MESA)

Whole genome sequencing (WGS) for the Trans-Omics in Precision Medicine (TOPMed) program was supported by the National Heart, Lung and Blood Institute (NHLBI). WGS for "NHLBI TOPMed: Multi-Ethnic Study of Atherosclerosis (MESA)" (phs001416.v3.p1) was performed at the Broad Institute of MIT and Harvard (3U54HG003067-13S1). Centralized read mapping and genotype calling, along with variant quality metrics and filtering were provided by the TOPMed Informatics Research Center (3R01HL-117626-02S1). Phenotype harmonization, data management, sample-identity QC, and general study coordination, were provided by the TOPMed Data Coordinating Center (3R01HL-120393-02S1), and TOPMed MESA Multi-Omics (HHSN2682015000031/HSN26800004). The MESA projects are conducted and supported by the National Heart, Lung, and Blood Institute (NHLBI) in collaboration with MESA investigators. Support for the Multi-Ethnic Study of Atherosclerosis (MESA) projects are conducted and supported by the National Heart, Lung, and Blood Institute (NHLBI) in collaboration with MESA investigators. Support for MESA is provided by contracts 75N92020D00001, HHSN268201500003I, N01-HC-95159, 75N92020D00005, N01-HC-95160, 75N92020D00002, N01-HC-95161, 75N92020D00003, N01-HC-95162, 75N92020D00006, N01-HC-95163, 75N92020D00004, N01-HC-95164, 75N92020D00007, N01-HC-95165, N01-HC-95166, N01-HC-95167, N01-HC-95168, N01-HC-95169, UL1-TR-000040, UL1-TR-001079, UL1-TR-001420, UL1TR001881, DK063491, and R01HL105756. The authors thank the other investigators, the staff, and the participants of the MESA study for their valuable contributions. A full list of participating MESA investigators and institutes can be found at http://www.mesa-nhlbi.org.

NHLBI TOPMed: My Life, Our Future (MLOF)

The My Life, Our Future samples and data are made possible through the partnership of Bloodworks Northwest, the American Thrombosis and Hemostasis Network, the National Hemophilia Foundation, and Bioverativ. We gratefully acknowledge the hemophilia treatment centers and their patients who provided biological samples and phenotypic data.

NHLBI TOPMed: Outcome Modifying Genes in Sickle Cell Disease (OMG-SCD)

The OMG-SCD study was administered by Marilyn J. Telen, M.D. and Allison E. Ashley-Koch, Ph.D. from Duke University Medical Center, and collection of the data set was supported by

grants HL068959 and HL079915 from the National Heart, Lung, and Blood Institute (NHLBI) of the National Institute of Health (NIH).

NHLBI TOPMed: Pediatric Cardiac Genomics Consortium's Congenital Heart Disease Biobank (PCGC-CHD)
The Pediatric Cardiac Genomics Consortium (PCGC) program is funded by the National Heart, Lung, and Blood Institute, National Institutes of Health, U.S. Department of Health and Human Services through grants UM1HL128711, UM1HL098162, UM1HL098147, UM1HL098123, UM1HL128761, and U01HL131003.

NHLBI TOPMed: The Pharmacogenomics of Hydroxyurea in Sickle Cell Disease (PharmHU)
Collection of the PharmHU samples and data were supported in part by the Department of Pediatrics, Baylor College of Medicine funds, National Institutes of Health (NIH) National Institute of Diabetes and Digestive and Kidney Diseases (NIDDK) grant 1K08 DK110448-01, NIH NHLBI R01 HL069234, and U01-HL117721 funded by NHLBI. We are very grateful to the patients with sickle cell disease for their participation in PharmHU.

NHLBI TOPMed: PIMA
This research was supported by grants from the National Heart, Lung, and Blood Institute (NHLBI), ((5U10HL064287, 5U10HL064288, 5U10HL064295, 5U10HL064307, 5U10HL064305, 5U10HL064313, and HL080083)

NHLBI TOPMed: PUSH_SCD
We thank Dr. Victor R Gordeuk and the investigators of the PUSH study and the patients who participated in the study. We also thank the PUSH clinical site team: Howard University: Victor R Gordeuk, Sergei Nekhai, Oswaldo Castro, Sohail Rana, Mehdi Nouraie, James G Taylor 6th, Children National Medical Center: Caterina Minniti, Deepika Darbari, Lori Lutchman-Jones, Nitti Dham, Craig Sable, NHLBI: Mark Gladwin, Greg Kato. University of Michigan: Andrew Campbell, Gregory Ensing, Manuel Arteta, Special thanks to the volunteers who participated in the PUSH study. This project was funded with federal funds from the NHLBI, NIH. Detail description of the study was published in Haematologica. 2009 Mar;94(3):340-7, MInniti C, et al. “Elevated tricuspid regurgitant jet velocity in children and adolescents with sickle cell disease: association with hemolysis and hemoglobin oxygen desaturation.”

NHLBI TOPMed: Recipient Epidemiology and Donor Evaluation Study-III (REDS-III_Brazil)
The Recipient Epidemiology and Donor Evaluation Study (REDS)-III was funded by NIH NHLBI contract HHSN268201100007I and conducted under the leadership of Simone Glynn (NHLBI), and principle investigators Brian Custer and Ester Sabino. We are grateful to the Brazilian sickle cell disease patients who participated in the REDS-III study and provided blood samples for whole genome sequencing as well as the REDS-III staff: Vitalant Research Institute (Shannon Kelly), University of Sao Paulo (Miriam V Flor Park, Ligia Capuani), Hemominas Belo Horizonte (Anna Barbara Proietti), Hemominas Montes Claros (Rosimere Alfonso), Hemominas Juiz de

Fora (Daniela de O. Werneck Rodrigues), Hemope (Paula Loureiro), Hemorio (Claudia Maximo).

NHLBI TOPMed: San Antonio Family Heart Study (SAFS)
Collection of the San Antonio Family Study data was supported in part by National Institutes of Health (NIH) grants P01 HL045522, R01 MH078143, R01 MH078111 and R01 MH083824; and whole genome sequencing of SAFS subjects was supported by U01 DK085524 and R01 HL113323. We are very grateful to the participants of the San Antonio Family Study for their continued involvement in our research programs.

NHLBI TOPMed: Study of African Americans, Asthma, Genes and Environment (SAGE)
The Study of African Americans, Asthma, Genes and Environments (SAGE) was supported by by the National Heart, Lung, and Blood Institute of the National Institute of Health (NIH) grants R01HL117004 and X01HL134589; study enrollment supported by the Sandler Family Foundation, the American Asthma Foundation, the RWJF Amos Medical Faculty Development Program, Harry Wm. and Diana V. Hind Distinguished Professor in Pharmaceutical Sciences II. The SAGE study collaborators include Harold J. Farber, Texas Children's Hospital; Emerita Brigino-Buenaventura, Kaiser Permanente; Michael A. LeNoir, Bay Area Pediatrics; Kelley Meade, UCSF Benioff Children’s Hospital, Oakland; Luisa N. Borrell, City University of New York; Adam Davis, UCSF Benioff Children’s Hospital, Oakland and Fred Lurmann, Sonoma Technologies, Inc. The authors acknowledge the families and patients for their participation and thank the numerous health care providers and community clinics for their support and participation in SAGE. In particular, the authors thank study coordinator Sandra Salazar; the recruiters who obtained the data: Lisa Caine, Elizabeth Castellanos, Brenda Lopez, MD, Shahdad Saeedi; and the lab researcher Celeste Eng who processed the biospecimens.

NHLBI TOPMed: Study of Asthma Phenotypes & Pharmacogenomic Interactions by RaceEthnicity (SAPPHIRE_asthma)
The SAPPHIRE cohort was supported by grant funding from the Fund for Henry Ford Hospital, the American Asthma Foundation, and the following institutes of the National Institutes of Health: the National Heart Lung and Blood Institute (R01HL141845, R01HL118267, X01HL134589, R01HL079055), the National Institute of Allergy and Infectious Diseases (R01AI079139, R01AI061774), and the National Institute of Diabetes and Digestive and Kidney Diseases (R01DK113003, R01DK064695).

NHLBI TOPMed: Genetics of Sarcoidosis in African Americans (Sarcoidosis)
Supported by the National Institutes of Health under Grant R01HL113326-05, P30 GM110766-01, and U54GM104938-06.

NHLBI TOPMed: Severe Asthma Research Program (SARP)
The authors acknowledge the contributions of the study coordinators and staff at each of the clinical centers and the Data Coordinating Center as well as all the study participants that have been integral to the success of the NHLBI Severe Asthma Research Program (funded by U10

HL109164, U10 HL109257, U10 HL109146, U10 HL109172, U10 HL109250, U10 HL109250, U10 HL109250, U10 HL109168, U10 HL109152, U10 HL109086).

NHLBI TOPMed: Genome-wide Association Study of Adiposity in Samoans (SAS)
Financial support from the U.S. National Institutes of Health Grants R01-HL093093 and R01HL133040. We acknowledge the assistance of the Samoa Ministry of Health and the Samoa Bureau of Statistics for their guidance and support in the conduct of this study. We thank the local village officials for their help and the participants for their generosity. The following publication describes the origin of the dataset: Hawley NL, Minster RL, Weeks DE, Viali S, Reupena MS, Sun G, Cheng H, Deka R, McGarvey ST. Prevalence of Adiposity and Associated Cardiometabolic Risk Factors in the Samoan Genome-Wide Association Study. Am J Human Biol 2014. 26: 491-501. DOI: 10.1002/jhb.22553. PMID: 24799123.

NHLBI TOPMed: Rare Variants for Hypertension in Taiwan Chinese (THRV)
The Rare Variants for Hypertension in Taiwan Chinese (THRV) is supported by the National Heart, Lung, and Blood Institute (NHLBI) grant (R01HL111249) and its participation in TOPMed is supported by an NHLBI supplement (R01HL111249-04S1). THRV is a collaborative study between Washington University in St. Louis, LA BioMed at Harbor UCLA, University of Texas in Houston, Taichung Veterans General Hospital, Taipei Veterans General Hospital, Tri-Service General Hospital, National Health Research Institutes, National Taiwan University, and Baylor University. THRV is based (substantially) on the parent SAPPHIRe study, along with additional population-based and hospital-based cohorts. SAPPHIRe was supported by NHLBI grants (U01HL54527, U01HL54498) and Taiwan funds, and the other cohorts were supported by Taiwan funds.

NHLBI TOPMed: The Vanderbilt AF Ablation Registry (VAFAR)
The research reported in this article was supported by grants from the American Heart Association to Dr. Shoemaker (11CRP742009), Dr. Darbar (EIA 0940116N), and grants from the National Institutes of Health (NIH) to Dr. Darbar (R01 HL092217), and Dr. Roden (U19 HL65962, and UL1 RR024975). The project was also supported by a CTSA award (UL1 TR00045) from the National Center for Advancing Translational Sciences. Its contents are solely the responsibility of the authors and do not necessarily represent the official views of the National Center for Advancing Translational Sciences or the NIH.

NHLBI TOPMed: The Vanderbilt Atrial Fibrillation Registry (VU_AF)
The research reported in this article was supported by grants from the American Heart Association to Dr. Darbar (EIA 0940116N), and grants from the National Institutes of Health (NIH) to Dr. Darbar (HL092217), and Dr. Roden (U19 HL65962, and UL1 RR024975). This project was also supported by CTSA award (UL1TR000445) from the National Center for Advancing Translational Sciences. Its contents are solely the responsibility of the authors and do not necessarily represent the official views of the National Center for Advancing Translational Sciences of the NIH.

NHLBI TOPMed: Treatment of Pulmonary Hypertension and Sickle Cell Disease With Sildenafil Therapy (Walk-PHaSST)
We thank Dr. Mark Gladwin and the investigators of the Walk-PHasst study and the patients who participated in the study. We also thanks the walk-PHaSST clinical site team: Albert Einstein College of Medicine: Jane Little and Verlene Davis; Columbia University: Robyn Barst, Erika Rosenzweig, Margaret Lee and Daniela Brady; UCSF Benioff Children's Hospital Oakland: Claudia Morris, Ward Hagar, Lisa Lavrisha, Howard Rosenfeld, and Elliott Vichinsky; Children's Hospital of Pittsburgh of UPMC: Regina McCollum; Hammersmith Hospital, London: Sally Davies, Gaia Mahalingam, Sharon Meehan, Ofelia Lebanto, and Ines Cabrita; Howard University: Victor Gordeuk, Oswaldo Castro, Onyinye Onyekwere,, Alvin Thomas, Gladys Onojobi, Sharmin Diaz, Margaret Fadojutimi-Akinsiku, and Randa Aladdin; Johns Hopkins University: Reda Girgis, Sophie Lanzkron and Durrant Barasa; NHLBI: Mark Gladwin, Greg Kato, James Taylor, Vandana Sachdev, Wynona Coles, Catherine Seamon, Mary Hall, Amy Chi, Cynthia Brenneman, Wen Li, and Erin Smith; University of Colorado: Kathryn Hassell, David Badesch, Deb McCollister and Julie McAfee; University of Illinois at Chicago: Dean Schraufnagel, Robert Molokie, George Kondos, Patricia Cole-Saffold, and Lani Krauz; National Heart & Lung Institute, Imperial College London: Simon Gibbs. Thanks also to the data coordination center team from Rho, Inc.: Nancy Yovetich, Rob Woolson, Jamie Spencer, Christopher Woods, Karen Kesler, Vickie Coble, and Ronald W. Helms. We also thank Dr. Yingze Zhang for directing the Walk-PHasst repository and Dr. Mehdi Nouraie for maintaining the Walk-PHasst database and Dr. Jonathan Goldsmith as a NIH program director for this study. Special thanks to the volunteers who participated in the Walk-PHaSST study. This project was funded with federal funds from the NHLBI, NIH, Department of Health and Human Services, under contract HHSN268200617182C. This study is registered at www.clinicaltrials.gov as NCT00492531. Detail description of the study was published in Blood, 2011 118:855-864, Machado et al "Hospitalization for pain in patients with sickle cell disease treated with sildenafil for elevated TRV and low exercise capacity".

NHLBI TOPMed: Novel Risk Factors for the Development of Atrial Fibrillation in Women (WGHS)
The WGHS is supported by the National Heart, Lung, and Blood Institute (HL043851 and HL080467) and the National Cancer Institute (CA047988 and UM1CA182913). The most recent cardiovascular endpoints were supported by ARRA funding HL099355.

NHLBI TOPMed: Women's Health Initiative (WHI)
The WHI program is funded by the National Heart, Lung, and Blood Institute, National Institutes of Health, U.S. Department of Health and Human Services through contracts HHSN268201600018C, HHSN268201600001C, HHSN268201600002C, HHSN268201600003C, and HHSN268201600004C. This manuscript was prepared in collaboration with investigators of the WHI, and has been reviewed and/or approved by the Women's Health Initiative (WHI). The short list of WHI investigators can be found at https://www.whi.org/researchers/Documents%20%20Write%20a%20Paper/WHI%20Investigator%20Sho rt%20List.pdf.

TOPMed Consortium members

Namiko Abe, Gonçalo Abecasis, Francois Aguet, Christine Albert, Laura Almasy, Alvaro Alonso, Seth Ament, Peter Anderson, Pramod Anugu, Deborah Applebaum-Bowden, Kristin Ardlie, Dan Arking, Donna K Arnett, Allison Ashley-Koch, Stella Aslibekyan, Tim Assimes, Paul Auer, Dimitrios Avramopoulos, Najib Ayas, Adithya Balasubramanian, John Barnard, Kathleen Barnes, R. Graham Barr, Emily Barron-Casella, Lucas Barwick, Terri Beaty, Gerald Beck, Diane Becker, Lewis Becker, Rebecca Beer, Amber Beitelshees, Emelia Benjamin, Takis Benos, Marcos Bezerra, Larry Bielak, Joshua Bis, Thomas Blackwell, John Blangero, Nathan Blue, Eric Boerwinkle, Donald W. Bowden, Russell Bowler, Jennifer Brody, Ulrich Broeckel, Jai Broome, Deborah Brown, Karen Bunting, Esteban Burchard, Carlos Bustamante, Erin Buth, Brian Cade, Jonathan Cardwell, Vincent Carey, Julie Carrier, April P. Carson, Cara Carty, Richard Casaburi, Juan P Casas Romero, James Casella, Peter Castaldi, Mark Chaffin, Christy Chang, Yi-Cheng Chang, Daniel Chasman, Sameer Chavan, Bo-Juen Chen, Wei-Min Chen, Yii-Der Ida Chen, Michael Cho, Seung Hoan Choi, Lee-Ming Chuang, Mina Chung, Ren-Hua Chung, Clary Clish, Suzy Comhair, Matthew Conomos, Elaine Cornell, Adolfo Correa, Carolyn Crandall, James Crapo, L. Adrienne Cupples, Joanne Curran, Jeffrey Curtis, Brian Custer, Coleen Damcott, Dawood Darbar, Sean David, Colleen Davis, Michelle Daya, Mariza de Andrade, Lisa de las Fuentes, Paul de Vries, Michael DeBaun, Ranjan Deka, Dawn DeMeo, Scott Devine, Huyen Dinh, Harsha Doddapaneni, Qing Duan, Shannon Dugan-Perez, Ravi Duggirala, Jon Peter Durda, Susan K. Dutcher, Charles Eaton, Lynette Ekunwe, Adel El Boueiz, Patrick Ellinor, Leslie Emery, Serpil Erzurum, Charles Farber, Jesse Farek, Tasha Fingerlin, Matthew Flickinger, Myriam Fornage, Nora Franceschini, Chris Frazar, Mao Fu, Stephanie M. Fullerton, Lucinda Fulton, Stacey Gabriel, Weiniu Gan, Shanshan Gao, Yan Gao, Margery Gass, Heather Geiger, Bruce Gelb, Mark Geraci, Soren Germer, Robert Gerszten, Auyon Ghosh, Richard Gibbs, Chris Gignoux, Mark Gladwin, David Glahn, Stephanie Gogarten, Da-Wei Gong, Harald Goring, Sharon Graw, Kathryn J. Gray, Daniel Grine, Colin Gross, C. Charles Gu, Yue Guan, Xiuqing Guo, Namrata Gupta, Jeff Haessler, Michael Hall, Yi Han, Patrick Hanly, Daniel Harris, Nicola L. Hawley, Jiang He, Ben Heavner, Susan Heckbert, Ryan Hernandez, David Herrington, Craig Hersh, Bertha Hidalgo, James Hixson, Brian Hobbs, John Hokanson, Elliott Hong, Karin Hoth, Chao (Agnes) Hsiung, Jianhong Hu, Yi-Jen Hung, Haley Huston, Chii Min Hwu, Marguerite Ryan Irvin, Rebecca Jackson, Deepti Jain, Cashell Jaquish, Jill Johnsen, Andrew Johnson, Craig Johnson, Rich Johnston, Kimberly Jones, Hyun Min Kang, Robert Kaplan, Sharon Kardia, Shannon Kelly, Eimear Kenny, Michael Kessler, Alyna Khan, Ziad Khan, Wonji Kim, John Kimoff, Greg Kinney, Barbara Konkle, Charles Kooperberg, Holly Kramer, Christoph Lange, Ethan Lange, Leslie Lange, Cathy Laurie, Cecelia Laurie, Meryl LeBoff, Jiwon Lee, Sandra Lee, Wen-Jane Lee, Jonathon LeFaive, David Levine, Daniel Levy, Joshua Lewis, Xiaohui Li, Yun Li, Henry Lin, Honghuang Lin, Xihong Lin, Simin Liu, Yongmei Liu, Yu Liu, Ruth J.F. Loos, Steven Lubitz, Kathryn Lunetta, James Luo, Ulysses Magalang, Michael Mahaney, Barry Make, Ani Manichaikul, Alisa Manning, JoAnn Manson, Lisa Martin, Melissa Marton, Susan Mathai, Rasika Mathias, Susanne May, Patrick McArdle, Merry-Lynn McDonald, Sean McFarland, Stephen McGarvey, Daniel McGoldrick, Caitlin McHugh, Becky McNeil, Hao Mei, James Meigs, Vipin Menon, Luisa Mestroni, Ginger Metcalf, Deborah A Meyers, Emmanuel Mignot, Julie Mikulla, Nancy Min, Mollie Minear, Ryan L Minster, Braxton D. Mitchell, Matt Moll,

Zeineen Momin, May E. Montasser, Courtney Montgomery, Donna Muzny, Josyf C Mychaleckyj, Girish Nadkarni, Rakhi Naik, Take Naseri, Pradeep Natarajan, Sergei Nekhai, Sarah C. Nelson, Bonnie Neltner, Caitlin Nessner, Deborah Nickerson, Osuji Nkechinyere, Kari North, Jeff O'Connell, Tim O'Connor, Heather Ochs-Balcom, Geoffrey Okwuonu, Allan Pack, David T. Paik, Nicholette Palmer, James Pankow, George Papanicolaou, Cora Parker, Gina Peloso, Juan Manuel Peralta, Marco Perez, James Perry, Ulrike Peters, Patricia Peyser, Lawrence S Phillips, Jacob Pleiness, Toni Pollin, Wendy Post, Julia Powers Becker, Meher Preethi Boorgula, Michael Preuss, Bruce Psaty, Pankaj Qasba, Dandi Qiao, Zhaohui Qin, Nicholas Rafaels, Laura Raffield, Mahitha Rajendran, Vasan S. Ramachandran, D.C. Rao, Laura Rasmussen-Torvik, Aakrosh Ratan, Susan Redline, Robert Reed, Catherine Reeves, Elizabeth Regan, Alex Reiner, Muagututi'a Sefuiva Reupena, Ken Rice, Stephen Rich, Rebecca Robillard, Nicolas Robin, Dan Roden, Carolina Roselli, Jerome Rotter, Ingo Ruczinski, Alexi Runnels, Pamela Russell, Sarah Ruuska, Kathleen Ryan, Ester Cerdeira Sabino, Danish Saleheen, Shabnam Salimi, Sejal Salvi, Steven Salzberg, Kevin Sandow, Vijay G. Sankaran, Jireh Santibanez, Karen Schwander, David Schwartz, Frank Sciurba, Christine Seidman, Jonathan Seidman, Frédéric Sériès, Vivien Sheehan, Stephanie L. Sherman, Amol Shetty, Aniket Shetty, Wayne Hui-Heng Sheu, M. Benjamin Shoemaker, Brian Silver, Edwin Silverman, Robert Skomro, Albert Vernon Smith, Jennifer Smith, Josh Smith, Nicholas Smith, Tanja Smith, Sylvia Smoller, Beverly Snively, Michael Snyder, Tamar Sofer, Nona Sotoodehnia, Adrienne M. Stilp, Garrett Storm, Elizabeth Streeten, Jessica Lasky Su, Yun Ju Sung, Jody Sylvia, Adam Szpiro, Daniel Taliun, Hua Tang, Margaret Taub, Kent D. Taylor, Matthew Taylor, Simeon Taylor, Marilyn Telen, Timothy A. Thornton, Machiko Threlkeld, Lesley Tinker, David Tirschwell, Sarah Tishkoff, Hemant Tiwari, Catherine Tong, Russell Tracy, Michael Tsai, Dhananjay Vaidya, David Van Den Berg, Peter VandeHaar, Scott Vrieze, Tarik Walker, Robert Wallace, Avram Walts, Fei Fei Wang, Heming Wang, Jiongming Wang, Karol Watson, Jennifer Watt, Daniel E. Weeks, Joshua Weinstock, Bruce Weir, Scott T Weiss, Lu-Chen Weng, Jennifer Wessel, Cristen Willer, Kayleen Williams, L. Keoki Williams, Scott Williams, Carla Wilson, James Wilson, Lara Winterkorn, Quenna Wong, Baojun Wu, Joseph Wu, Huichun Xu, Lisa Yanek, Ivana Yang, Ketian Yu, Seyedeh Maryam Zekavat, Yingze Zhang, Snow Xueyan Zhao, Wei Zhao, Xiaofeng Zhu, Elad Ziv, Michael Zody, Sebastian Zoellner

Hematology and Hemostasis working group members

Laura Almasy, Kurtis Anthony, Dan Arking, Allison Ashley-Koch, Paul Auer, Abraham Aviv, Andrea Baccarelli, Emily Barron-Casella, Lewis Becker, Romit Bhattacharya, Alexander Bick, Larry Bielak, Thomas Blackwell, John Blangero, Kelly Bolton, Jennifer Brody, Derek Brown, Deepika Burkardt, James Casella, Liam Cato, Christy Chang, Nilanjan Chatterjee, Han Chen, Ming-Huei Chen, Michael Cho, Zeynep Coban Akdemir, Jason Collins, Karen Conneely, Matthew Conomos, Paul de Vries, Dawn DeMeo, Pinkal Desai, Qing Duan, Connor Emdin, Nauder Faraday, Annette Fitzpatrick, Travis Fleming, James Floyd, Santhi Ganesh, Brady Gaynor, LaShaunta Glover, Jacob Graham, Edward Ha, Nadia Hansel, Manjit Hanspal, Ross Hardison, Ben Heavner, Julian Hecker, Scott Heemann, Craig Hersh, Chani Hodonsky, Michael Honigberg, Steve Horvath, Yao Hu, Jennifer Huffman, Carmen Isasi, Kruthika Raman Iyer, Sidd Jaiswal, Cashell Jaquish, Jin Jin, Jill Johnsen, Andrew Johnson, Brian Joyce, Joel Kaufman, Rebecca Keener, Shannon Kelly, Alyna Khan, Sumeet Khetarpal, Greg Kinney, Malgorzata

Klauzinska, Barbara Konkle, Charles Kooperberg, Mohanraj Krishnan, Ethan Lange, Leslie Lange, Cathy Laurie, Brandon Lê, Grace Lee, Claire Leiser, Guillaume Lettre, Dan Levy, Joshua Lewis, Bingshan Li, Yun Li, L. A. Liggett, Amarise Little, Shelly-Ann Love, Megan Lynch, Mitchell Machiela, Rasika Mathias, Ravi Mathur, Karen Miga, Anna Mikhaylova, Julie Mikulla, Braxton D. Mitchell, Alanna C Morrison, Rakhi Naik, Drew Nannini, Vivek Naranbhai, Pradeep Natarajan, Jeff O'Connell, Christopher O'Donnell, Nels Olson, Helena Palma Gudiel, Nathan Pankratz, Benedict Paten, James Perry, James Pirruccello, Linda Polfus, Diddier Prada, Bruce Psaty, Laura Raffield, Elizabeth Regan, Alex Reiner, Stephen Rich, Shabnam Salimi, Vijay G. Sankaran, Noah Simon, Nicholas Smith, James Stewart, Adrienne M. Stilp, Shakira Suglia, Weihong Tang, Hua Tang, Margaret Taub, Kent D. Taylor, Marilyn Telen, Florian Thibord, Timothy A. Thornton, Russell Tracy, Md Mesbah Uddin, Heming Wang, Lachelle Weeks, Joshua Weinstock, Ellen Werner, Marsha Wheeler, Eric Whitsel, Kerri L. Wiggins, Lisa Yanek, Yu-Chung Yang, Kimberley Youkhana, Michael Young, Anthony Zannas, Seyedeh Maryam Zekavat, Wei Zhao, Yinan Zheng, Ying Zhou

Structural Variation working group members

Paul Auer, Kathleen Barnes, Thomas Blackwell, Harrison Brand, Ulrich Broeckel, Deepika Burkardt, Mark Chaisson, Kei Hang Katie Chan, Seung Hoan Choi, Zechen Chong, Bradley Coe, John Cole, Ryan Collins, Matthew Conomos, Michelle Daya, Scott Devine, Evan Eichler, Annette Fitzpatrick, C. Charles Gu, Amelia Weber Hall, Ira Hall, Bob Handsaker, Ben Heavner, Scott Heemann, James Hixson, Jicai Jiang, Jill Johnsen, Michelle Jones, Brian Joyce, Goo Jun, Hyun Min Kang, Spencer Kelley, Charles Kooperberg, John Lane, Cathy Laurie, Seung-been, Steven Lee, Dan Levy, Yang Li, Honghuang Lin, Simin Liu, Angel CY Mak, Alisa Manning, Rasika Mathias, Steve McCarroll, Julie Mikulla, Jean Monlong, Drew Nannini, Giuseppe Narzisi, Jeff O'Connell, Wanda O'Neal, Grier Page, Nathan Pankratz, Benedict Paten, Alexandre Pereira, Patricia Peyser, Nathan Pezant, Gloria Quach, Aakrosh Ratan, Alex Reiner, Stephen Rich, Ingo Ruczinski, Aniko Sabo, Steven Salzberg, Jonathan Seidman, Minseok Seo, Yichen Si, Nasa Sinnott Armstrong, Albert Vernon Smith, Vinodh Srinivasasainagendra, Arvis Sulovari, Margaret Taub, Joshua Weinstock, Marsha Wheeler, James Wilson, Huichun Xu, Wei Zhao, Xuefang Zhao, Yinan Zheng, Degui Zhi, Sebastian Zoellner
