# Supplementary material for: Validation of human telomere length multi-ancestry meta-analysis association signals identifies POP5 and KBTBD6 as human telomere length regulation genes
Source: Nat Commun. 2024 May 24;15:4417. doi: 10.1038/s41467-024-48394-y (PMC11126610; doi:10.1038/s41467-024-48394-y)
Supplement: Supplementary file 3 — Description of Additional Supplementary Files [file 41467_2024_48394_MOESM3_ESM.pdf]

Description of Additional Supplementary Files

File Name: Supplementary Data 1
Description: Cohorts used in either the age-stratified GWAS or meta-analysis.

File Name: Supplementary Data 2
Description: Summary statistics for lead SNPs at each GWAS meta-analysis signal.

File Name: Supplementary Data 3
Description: Comparison of GWAS meta-analysis lead SNPs across input cohorts (and TOPMed pooled analysis).

File Name: Supplementary Data 4
Description: All colocalization results with GTEx cis-eQTLs.

File Name: Supplementary Data 5
Description: All colocalization results with GTEx sQTLs.

File Name: Supplementary Data 6
Description: All colocalization results with eQTLGen cis-eQTLs.

File Name: Supplementary Data 7
Description: All colocalization results with DICE cis-eQTLs.

File Name: Supplementary Data 8
Description: SuSiE 95% credible set analysis for each meta-analysis signal.

File Name: Supplementary Data 9
Description: PANTHER GO Enrichment analysis.

File Name: Supplementary Data 10
Description: A list of transcription factors (TF) that were analyzed in the transcription factor binding site enrichment analysis.

File Name: Supplementary Data 11
Description: The lead SNP of each meta-analysis signal was intersected with the JASPAR 2022 track in the UCSC genome browser.

File Name: Supplementary Data 12
Description: Summary statistics for a GWAS on 109,122 TOPMed individuals including an interaction term between age and genotype.

File Name: Supplementary Data 13
Description: Age-stratified GWAS summary statistics.

File Name: Supplementary Data 14
Description: Roadmap Epigenomics samples that were used in the analysis of the 25 state chromHMM model (chromHMM_25state).

File Name: Supplementary Data 15
Description: Primer, sgRNA, and Southern probe sequences.
