# Supplementary material for: Validation of human telomere length multi-ancestry meta-analysis association signals identifies POP5 and KBTBD6 as human telomere length regulation genes
Source: Nat Commun. 2024 May 24;15:4417. doi: 10.1038/s41467-024-48394-y (PMC11126610; doi:10.1038/s41467-024-48394-y)
Supplement: Supplementary file 19 — Reporting Summary [file 41467_2024_48394_MOESM19_ESM.pdf]

nature portfolio

Corresponding author(s): Rasika Mathias and Alexis Battle

Last updated by author(s): March 8, 2024

Reporting Summary

Nature Portfolio wishes to improve the reproducibility of the work that we publish. This form provides structure for consistency and transparency in reporting. For further information on Nature Portfolio policies, see our Editorial Policies and the Editorial Policy Checklist.

Statistics

For all statistical analyses, confirm that the following items are present in the figure legend, table legend, main text, or Methods section.

n/a	Confirmed	
☐	☒	The exact sample size (n) for each experimental group/condition, given as a discrete number and unit of measurement
☐	☒	A statement on whether measurements were taken from distinct samples or whether the same sample was measured repeatedly
☐	☒	The statistical test(s) used AND whether they are one- or two-sided<br>*Only common tests should be described solely by name; describe more complex techniques in the Methods section.*
☐	☒	A description of all covariates tested
☐	☒	A description of any assumptions or corrections, such as tests of normality and adjustment for multiple comparisons
☐	☒	A full description of the statistical parameters including central tendency (e.g. means) or other basic estimates (e.g. regression coefficient) AND variation (e.g. standard deviation) or associated estimates of uncertainty (e.g. confidence intervals)
☐	☒	For null hypothesis testing, the test statistic (e.g. F, t, r) with confidence intervals, effect sizes, degrees of freedom and P value noted<br>*Give P values as exact values whenever suitable.*
☐	☒	For Bayesian analysis, information on the choice of priors and Markov chain Monte Carlo settings
☒	☐	For hierarchical and complex designs, identification of the appropriate level for tests and full reporting of outcomes
☐	☒	Estimates of effect sizes (e.g. Cohen's d, Pearson's r), indicating how they were calculated

Our web collection on statistics for biologists contains articles on many of the points above.

Software and code

Policy information about availability of computer code

Data collection	No software was used to for data collection in these experiments.
Data analysis	Meta-analysis was conducted using GWAMA. To analyze western and Southern blot data we used ImageQuant TL and Adobe PhotoShop CS6.<br>To identify candidate CRISPR/Cas9 guides we used CRISPOR.org.<br>In the course of this work we used the following software: R (3.5.1), SAMtools (1.16), Matplotlib, CAVIAR (3.6.5), PANTHER (2022-07-01), BEDTools (2.29.2), Analysis Commons, S-LDSC (1.0.1), Snapgene (5.0.8), FUSION<br>In the course of this work we used the following R packages: data.table (1.14.6), rtracklayer, ggplot2 (3.4.0), coloc, dplyr (1.1.0), susieR (0.12.16), stringr (1.5.0), cowplot (1.1.1), ggVennDiagram (1.2.2), devtools (2.4.5), usethis (2.1.6), scales (1.2.1), ggrepel (0.9.3), patchwork (1.1.2)<br>Code related to this manuscript can be found on GitHub:<br>https://github.com/RKeener/telomere_length_metaanalysis<br>https://github.com/BennyStrobes/leafcutter_sqtl_viz<br>Code and dependent files used to reproduce figures can be found on Zenodo:<br>doi: 10.5281/zenodo.10476185<br>Summary statistics, plasmid maps, unprocessed blot images, and analysis dependent files are available at Zenodo<br>(doi: 10.5281/zenodo.10476137)

For manuscripts utilizing custom algorithms or software that are central to the research but not yet described in published literature, software must be made available to editors and reviewers. We strongly encourage code deposition in a community repository (e.g. GitHub). See the Nature Portfolio guidelines for submitting code & software for further information.

Data

Policy information about availability of data

All manuscripts must include a data availability statement. This statement should provide the following information, where applicable:

- Accession codes, unique identifiers, or web links for publicly available datasets
- A description of any restrictions on data availability
- For clinical datasets or third party data, please ensure that the statement adheres to our policy

All cell lines and plasmids are available upon request. Summary statistics, plasmid maps, unprocessed blot images, and analysis dependent files are available at Zenodo (doi: 10.5281/zenodo.10476137) and are freely available. TOPMed genomic data and telomere length estimates are available by study in the database of Genotypes and Phenotypes (dbGaP) (https://www.ncbi.nlm.nih.gov/gap/?term=TOPMed). GTEx_v8 eQTL, sQTL, and LeafCutter exon-exon junction quantifications are available for download through the GTEx portal (https://gtexportal.org/home/). eQTLGen cis-eQTL data are available for download (https://www.eqtlgen.org/). In this manuscript we used the version available 2019-12-11. DICE cis-eQTL data are available for download (https://dice-database.org/landing). In this manuscript we used the version available 2019-06-07. Roadmap Epigenomics data can be visualized and downloaded here: https://egg2.wustl.edu/roadmap/web_portal/. ATAC-seq downloaded from ENCODE can be found here: https://www.encodeproject.org/. ATACdb data can be downloaded here: https://bio.tools/atacdb. ENCODE transcription factor ChIP-seq track data can be downloaded here (340 factors in 129 cell types from ENCODE 3): https://genome.ucsc.edu/cgi-bin/hgTrackUi?hgsid=1997834034_KyrOSyi5TZL4ybD9G2z2TOU6TCkR&c=chr7&g=encTfChipPk. ReMap 2022 data can be downloaded here: https://remap2022.univ-amu.fr/about_hsap_page. JASPAR 2022 transcription factor binding site data can be downloaded here: https://genome.ucsc.edu/cgi-bin/hgTrackUi?hgsid=1997834034_KyrOSyi5TZL4ybD9G2z2TOU6TCkR&db=hg38&c=chr7&g=jaspar.

Research involving human participants, their data, or biological material

Policy information about studies with human participants or human data. See also policy information about sex, gender (identity/presentation), and sexual orientation and race, ethnicity and racism.

Reporting on sex and gender	Biological sex was a covariate in the GWAS linear regression model for the age stratified GWAS and GWAS that included an interaction term between age and genotype. Findings apply to both biological sexes. Information on biological sex for each individual can be accessed through the TOPMed dbGaP exchange area.
Reporting on race, ethnicity, or other socially relevant groupings	In a previous study, Taub et al. 2022, TOPMed individuals were stratified by ancestry group where individuals were broadly categorized as European, African, Asian, or Hispanic/Latino using HARE and we maintain language used from that study here for clarity. A GWAS was conducted on each ancestry group and we meta-analyzed these GWAS in addition to data from three other studies in our meta-analysis. The three other studies reported that they recruited individuals from specific regions and described their populations as White European, Bangladeshi, or Singaporean Chinese. In our text we refer to "Asian" broadly as including TOPMed Asian, the Bangladeshi GWAS, and the Singaporean Chinese GWAS, but we did not combine these studies prior to meta-analysis with the other GWAS.<br>Only TOPMed individuals were included in the age-stratified GWAS and in the GWAS that included an interaction term between age and genotype. In these analyses, the HARE derived ancestry was included as a covariate in the GWAS.
Population characteristics	We will limit our description to TOPMed individuals as that was the only individual-level data used in this study. The age range is 0-98 years old, it includes both males and females, individuals are a mixture of healthy and diagnosed with various heart, lung, blood, and sleep disorders. Whole genome sequencing was harmonized across all individuals in TOPMed.
Recruitment	We did not recruit any individuals in the course of this work.
Ethics oversight	Our study was approved by the TOPMed Hemotology and Hemostasis working group, in addition to the TOPMed Analysis Committee Chairs.

Note that full information on the approval of the study protocol must also be provided in the manuscript.

Field-specific reporting

Please select the one below that is the best fit for your research. If you are not sure, read the appropriate sections before making your selection.

☒ Life sciences ☐ Behavioural & social sciences ☐ Ecological, evolutionary & environmental sciences

For a reference copy of the document with all sections, see nature.com/documents/nr-reporting-summary-flat.pdf

Life sciences study design

All studies must disclose on these points even when the disclosure is negative.

Sample size	For the age-stratified GWAS and the GWAS that included an interaction term between age and genotype, we included 109,122 TOPMed individuals. This number was chosen as the maximum number of individuals for whom a telomere length estimate was available.<br>For the telomere length Southern blot experiments it is standard in the field to examine three clones as clonal experiments of telomere length as measured by Southern blot can be significantly distinct between clones. Some studies examine a single clone but it is widely accepted that included multiple clones with similar telomere length effects enables much higher confidence in the conclusions. This practice was used in seminal publications in the field, such as Smogorzewska et al. 2000 (PMID: 10669743). We passaged them for at least 200 population doublings to observe changes in telomere length as is standard in the field, as previously demonstrated in van Steensel and de Lange 1997

(PMID: 9034193).
For the CRISPR/Cas9 editing experiments we generated as many clones as possible, evaluating a total of 750 clones across all CRISPR experiments in this study. Some studies use as few as three or four biological replicates for such analyses, for example, PMID: 38417378, PMID: 38365720.
For LDSC, TWAS, SuSiE, and CAVIAR we met the recommended sample size for each analysis.

Data exclusions

No data were excluded from our analyses

Replication

We identified five novel telomere length associated signals, demonstrating that the vast majority of our signals are replicated. Furthermore, replication analysis of our meta-analysis summary statistics with an independent telomere length GWAS allowed us to examine four of our novel signals for replication, of which two significantly replicated.
We conducted colocalization analysis across multiple independent QTL datasets and report the replication of colocalization for each meta-analysis signal. Furthermore, we discuss the convergence of colocalization analysis, proximal genes, and TWAS results in Supplementary Note 1.
We used multiple clones for our overexpression experiment to demonstrate that changes on protein levels and telomere length were consistent across biological replicates. Samples were run on multiple Southern blots and western blots and while there was technical variation in the quality of the blot (some ran slightly crooked, exposure was not as clean) the conclusions were replicated across all attempts.
In our qPCR analysis we ran each sample with three technical replicates and each concentration of the standard was done in duplicate. If the standard line of best fit was less than 0.98 that plate was not used. If the range of Cq for technical replicates of a sample was greater than 1, we did not use those results and repeated the qPCR. All qPCR analysis that met these criteria were included in the dataset described in this study and were successful for the conclusions made using this data.

Randomization

Randomization is relevant for our qPCR samples where we ensured that each plate had a combination of control and experimental samples in addition to the standard curve samples. We examined whether there were batch effects across plates and did not observe a significant pattern therefore it was not relevant to attempt to adjust the qPCR results by batch for our analysis.

Blinding

Blinding is not relevant for association testing as the covariates must be observed to conduct the experiment. For the Southern blot samples the researcher handling the cells and generating the Southern blots was not blinded but the lead investigators were blinded during data interpretation to provide an unbiased interpretation of the results.

Reporting for specific materials, systems and methods

We require information from authors about some types of materials, experimental systems and methods used in many studies. Here, indicate whether each material, system or method listed is relevant to your study. If you are not sure if a list item applies to your research, read the appropriate section before selecting a response.

Materials & experimental systems

n/a	Involved in the study	
☐	☒	Antibodies
☐	☒	Eukaryotic cell lines
☒	☐	Palaeontology and archaeology
☒	☐	Animals and other organisms
☒	☐	Clinical data
☒	☐	Dual use research of concern
☒	☐	Plants

Methods

n/a	Involved in the study	
☒	☐	ChIP-seq
☒	☐	Flow cytometry
☒	☐	MRI-based neuroimaging

Antibodies

Antibodies used

M2 FLAG (Sigma F1804-5MG), beta tubulin (Abcam ab6046), HRP conjugated goat anti mouse secondary (BioRad 170-6516), and HRP-anti rabbit secondary (BioRad 170-6515).

Validation

M2 FLAG validation by Sigma: https://www.sigmaaldrich.com/deepweb/assets/sigmaaldrich/product/documents/754/849/anti-flag-2poster.pdf
beta tubulin validation from Abcam: https://www.abcam.com/products/primary-antibodies/beta-tubulin-antibody-loading-control-ab6046.html
Goat anti-mouse HRP conjugated secondary antibody from BioRad: https://www.bio-rad-antibodies.com/polyclonal/mouse-igg-antibody-star207.html?f=hrp
Citations validating the use of Goat anti-rabbit HRP-conjugated secondary antibody from BioRad are available here: https://www.bio-rad-antibodies.com/polyclonal/rabbit-lapine-igg-antibody-star124.html?f=hrp

Eukaryotic cell lines

Policy information about cell lines and Sex and Gender in Research

Cell line source(s)

K562 cells (female) were purchased from the ATCC. HeLa-FRT cells (female) were previously generated and stored by the Greider lab.

Authentication	No cell lines were authenticated
Mycoplasma contamination	Cell lines were not tested for mycoplasma contamination
Commonly misidentified lines (See ICLAC register)	None
